# Comparative proteomic analysis revealed complex responses to classical/novel duck reovirus infections in *Cairna moschata*

**DOI:** 10.1038/s41598-018-28499-3

**Published:** 2018-07-04

**Authors:** Tao Yun, Jionggang Hua, Weicheng Ye, Bin Yu, Liu Chen, Zheng Ni, Cun Zhang

**Affiliations:** Institute of Animal Husbandry and Veterinary Sciences, Zhejiang Academy of Agricultural Sciences, Hangzhou, 310021 China

## Abstract

Duck reovirus (DRV) is an typical aquatic bird pathogen belonging to the *Orthoreovirus* genus of the *Reoviridae* family. Reovirus causes huge economic losses to the duck industry. Although DRV has been identified and isolated long ago, the responses of *Cairna moschata* to classical/novel duck reovirus (CDRV/NDRV) infections are largely unknown. To investigate the relationship of pathogenesis and immune response, proteomes of *C*. *moschata* liver cells under the C/NDRV infections were analyzed, respectively. In total, 5571 proteins were identified, among which 5015 proteins were quantified. The differential expressed proteins (DEPs) between the control and infected liver cells displayed diverse biological functions and subcellular localizations. Among the DEPs, most of the metabolism-related proteins were down-regulated, suggesting a decrease in the basal metabolisms under C/NDRV infections. Several important factors in the complement, coagulation and fibrinolytic systems were significantly up-regulated by the C/NDRV infections, indicating that the serine protease-mediated innate immune system might play roles in the responses to the C/NDRV infections. Moreover, a number of molecular chaperones were identified, and no significantly changes in their abundances were observed in the liver cells. Our data may give a comprehensive resource for investigating the regulation mechanism involved in the responses of *C*. *moschata* to the C/NDRV infections.

## Introduction

Duck reovirus (DRV), a member of the genus *Orthoreovirus* in the family *Reoviridae*, is a fatal aquatic bird pathogen^1^. DRV was first identified in France (named MDRV), and then was isolated in Israel^1^, Italy^2^ and Germany^3^. In China, the disease caused by DRV was firstly reported in 1997^4^. MDRV (also called classical DRV, CDRV) mainly infected Muscovy ducklings. Diseases that were associated with CDRV infection showed a series of clinical symptoms, including general weakness, diarrhea, growth retardation, pericarditis, swollen liver and pleen covered with small white necrotic foci^5–7^. Recently, a novel duck reovirus (NDRV) was commonly identified in various duck species, such as Pekin duck, Cherry Valley duck and geese in China^8^. The principal lesions of the disease are hemorrhagic-necrotic lesions in liver and spleen^9^. To date, current commercial vaccines could not completely prevent the infection and transmission of C/NDRV^10^. Thus, many studies have focused on the regulation mechanisms underlying disease pathogenesis of DRVs.

DRVs contain 10 double-stranded RNA segments, including three large segments (named L1-3), three medium segments (named M1-3) and four small segments (named S1-4), according to their electrophoretic mobilities^11,12^. At least, 10 structural proteins and four nonstructural proteins are encoded by the DRV genome^13^.

DRV causes high mortality and morbidity in ducklings; however, the responses of Muscovy ducks to the DRV infection are largely unknown^14,15^. In the past years, a number of DRV infection responsive genes have been identified in ducklings. For example, a transcriptome profile of Muscovy duckling spleen cells pointed out that several genes involved in the innate immune system responded to the MDRV infection^14^. Another transcriptome revealed that the MDRV infection repressed the efflux of cholesterol from hepatic cells and down-regulated the expression of several fatty acid degradation-related genes^16^. These data have provided a base for the studies on the DRV infection in future.

So far, a few proteomic studies have been performed in ducks. A label-free LC-MS proteomic analysis of duck ovarian follicles revealed the host responses to the duck *tembusu* virus infection^17^. In Pekin duck, 54 differential expressed proteins (DEPs) responsive to heat stress were identified by MALDI-TOF/TOF-MS^18^. By 2-D gel electrophoresis, a total of 59 differentially expressed proteins with functions in the utilization of carbohydrates and nucleotides, stress defenses, and the regulation of immune system were indentified under MDRV infection^19^. Recently, a novel MS/MS-based analysis involving isotopomer labels and ‘tandem mass tags’ (TMTs), has been newly developed for protein accurate quantification^20^. Taking advantage of this newly developed technique, a large number of DEPs under the C/NDRV infections were identified in our study. The purpose of our study is investigation of the relationship of pathogenesis and immune response. The results will provide useful information on the pathogenicity of C/NDRV in ducks and new insights into the further studies on this disease.

## Materials and Methods

### Ethics statement

Animal experiment was conducted in the Institute of Animal Husbandry and Veterinary Sciences (IAHV), Zhejiang Academy of Agricultural Sciences (ZAAS). All ducks used in our experiments were treated in accordance with the Regulations of the Administration of Affairs Concerning Experimental Animals approved by the State Council of China. The bird protocol used in this study was approved by the Research Ethics Committee of ZAAS (permit number: ZAAS20160802).

### Experimental birds and virus

The experimental infection was conducted in 1-day-old healthy Muscovy ducklings, and the blood samples of each duckling were checked by RT-PCR to ensure their healthy (no DRV has been detected). Twenty seven 1-day-old Muscovy ducklings were randomly divided into three groups and were housed in the isolation facility. For the CDRV infection, one group (9 ducklings) was inoculated intramuscular with 0.5 mL of CDRV ZJ2000M strain at a titer of 10^5.19^ median tissue culture infective dose (TCID50) per mL. For the NDRV infection, another group (9 ducklings) was inoculated intramuscular with 0.5 mL of cell culture medium infected by NDRV HN10 strain contained the same TCID50, the titer of NDRV HN10 strain is 10^6.4^ TCID50 per mL. Additionally, nine un-infected ducklings were treated with sterile DMEM in the same manner. Ducklings of each groups were euthanatized after 72 hours post infection (hpi). Their livers were collected and snap-frozen in liquid nitrogen. Subsequently, frozen tissues were kept at − 80 °C until further processing.

### Protein extraction and trypsin digestion

For each treatment group, nine ducklings were divided into three independent subgroups (three ducklings as a repeat). Samples were first grinded by liquid N_2_ and then were transferred into a 5 mL tube. The cell powder was sonicated using a high intensity ultrasonic processor in pre-cooled lysis buffer containing 8 M urea, 2 mM ethylenediaminetetraacetic acid, 10 mM dithiothreitol and 1% Protease Inhibitor Cocktail VI. The remaining debris was discarded by centrifugation at 20,000 × g for 10 min at 4 °C. At last, the samples were precipitated with pre-cooled 15% TCA buffer for 2 h, and the supernatant was removed by centrifugation at 20,000 × g for 10 min at 4 °C. Again, the remaining precipitate was washed for three times with pre-cooled acetone and was redissolved in buffer containing 8 M urea, 100 mM TEAB (pH 8.0).

The resulting solution was reduced with 10 mM dithiothreitol at 37 °C and was alkylated with 20 mM iodoacetamide at 25 °C in darkness for 45 min. For trypsin digestion, the samples were digested with Trypsin Gold (Promega, Madison, WI, USA) to produce the digested peptides. The trypsin was added at a mass ratio of 1:50 (trypsin:protein) for a overnight digestion and at a mass ratio of 1:100 (trypsin: protein) for a 4 h digestion. Approximately 100 μg of each sample was digested for the further experiments.

### TMT labeling

The digested peptide samples were desalted though a Strata X-C18-SPE column (Phenomenex, Torrance, CA, USA) and vacuum-dried. For TMT labeling, peptides were reconstituted in 0.5 M triethylammonium bicarbonate buffer and processed using a 6-plex TMT kit according to the its protocol (Thermo-Scientific, Rockford, USA). Briefly, one unit of TMT reagent, which was added to label 100 μg of protein, was thawed and reconstituted in 24 μL acetonitrile. The peptide-reagent mixture was incubated at room temperature for 2 h, pooled, desalted, and dried by vacuum centrifugation.

### HPLC fractionation and LC-MS/MS analysis

The samples were crushed into various fractions by high pH reverse-phase HPLC with Agilent 300Extend C18 column (Santa Clara, CA, USA) at wavelength 250 nm. Briefly, peptide samples were fractioned into 80 fractions with a gradient of 2% to 60% acetonitrile in 10 mM ammonium bicarbonate for 80 min, at pH 10. Then, all fractions were combined into 18 fractions and were vacuum dried.

For LC-MS/MS analysis, peptides were dissolved in 0.1% formic acid and directly loaded onto an Acclaim PepMap 100 reversed-phase pre-column (ThermoFisher Scientific, Shanghai, China). Peptide separation was carried out using an Acclaim PepMap RSLC reversed-phase analytical column (ThermoFisher Scientific, Shanghai, China). The gradient was comprised of a lifting from 6% to 22% of solution B (0.1% FA in 98% ACN) in 22 min, an increase from 22% to 36% of solution B in 10 min, an increase from 36% to 85% of solution B in 5 min, and holding at 85% of solution B for 3 min with a steady flow rate of 400 nL/min on an EASY-nLC 1000 UPLC system (Thermo, Shanghai, China).

The resulting peptides were subjected to NSI source followed by MA/MA in Q Exactive^TM^ plus (Thermo, Shanghai, China) coupled online to the UPLC system. Intact peptides were detected in the Orbitrap at a resolution of 70,000 and ion fragments were detected at a resolution of 17,500. The applied electrospray voltage was set to 2.0 kV. Automatic gain control was applied to prevent overfilling of the ion trap and 5E4 ions were accumulated to generate the MS/MS spectra. The m/z scan range was set at 350 to 1800 for MS scans and the first fixed mass was set at 100 m/z.

### Database search

The resulting MS/MS data were processed using *Thermo Proteome Discoverer* (version 2.1.0.81) with mascot search engine against unip_Anas_8839 database. Trypsin/P was treated as cleavage enzyme allowing up to 2 missing cleavages, 5 modifications, and 5 charges per peptide. Mass error was set to 10 ppm for precursor ions and to 0.02 Da for fragment ions. Carbamido-methylation on cysteine, TMT-6-plex on lysine and N-term was treated as fixed modification, and oxidation on methionine was treated as variable modifications. The threshold for false discovery rates (FDRs) of protein, peptide and modification site was set at 0.01. Minimum peptide length was specified at 7. All the other parameters in *Thermo Proteome Discoverer* were set to default values.

The mass spectrometry proteomics data have been deposited to the ProteomeXchange Consortium via the PRIDE partner repository with the dataset identifier PXD008623.

### Bioinformatic analysis

Gene Ontology (GO) annotation of all identified proteins was derived from the UniProt-GOA database (http://www.ebi.ac.uk/GOA/). The IDs of all identified proteins were converted to the UniProt IDs, which could be mapped to the GO maps. The unannotated proteins were annotated by the InterProScan software by the protein sequence alignment method.

Kyoto Encyclopedia of Genes and Genomes (KEGG) database was used to annotate metabolic and biological processes (http://www.genome.jp/kegg/). KEGG description of each identified protein was annotated by a KEGG online service tool, KAAS. Then, all annotated proteins were mapped onto the KEGG pathway using KEGG mapper, another KEGG online service tool.

An updated version of PSORT/PSORT II was used to predict the subcellular localizations of all identified proteins (http://www.genscript.com/wolf-psort.html).

Protein domain was analyzed by a sequence analysis software, InterProScan, basing on protein sequence alignment method (http://www.ebi.ac.uk/interpro/).

### Enrichment analysis of the DEPs

GO, KEGG and proteins domain enrichments of the DEPs under C/NDRV infections were analyzed. For each GO, KEGG and domain category, a two-tailed Fisher’s exact test was carried out to determine the enrichments of the DEPs against the background (all identified proteins in this study). Correction for multiple hypothesis testing was employed using standard FDR control method. GO, KEGG or domain category with a *P* value** < **0.05 was treated significant. To satisfy the conditions of the hierarchical clustering method, the *P* values were transformed into Z-score:

$$Z\,sample-i=\frac{\mathrm{log}\,2({\rm{Signalsample}}-{\rm{i}})-{\rm{Mean}}(\mathrm{Log}\,2({\rm{Signal}}){\rm{of}}\,{\rm{all}}\,{\rm{samples}})}{{\rm{Standard}}\,\mathrm{deviation}\,(\mathrm{Log}\,2({\rm{Signal}}){\rm{of}}\,{\rm{all}}\,{\rm{samples}})}$$

### Analysis of protein-protein interaction (PPI) network

All DEPs under C/NDRV infections were searched against the STRING database version 10.0 for PPI predication. The PPIs that were belonged to our data sets were selected to exclude external candidate proteins. A metric ‘confidence score’ was used to define interaction confidence, and the interactions with a confidence score < 0.7 were fetched. Cytoscape (ver. 3.5.1) was used to visualize the PPI network from STRING database.

### Validation of DEPs by Parallel Reaction Monitoring (PRM)

The PRM assay was used to confirm the changes of DEPs identified in the LC–MS/MS-TMT analysis. Briefly, the methods of protein extraction and Trypsin digestion was the same as the description above. In PRM, peptides were identified by an acquired MS/MS spectrum. Sensitivity is highly related to peptide identification. Thus, peptide identification is essential, posing an inherent limit in data-dependent MS/MS scans.

## Result

### Quality validation of the MS data

**Figure 1:**
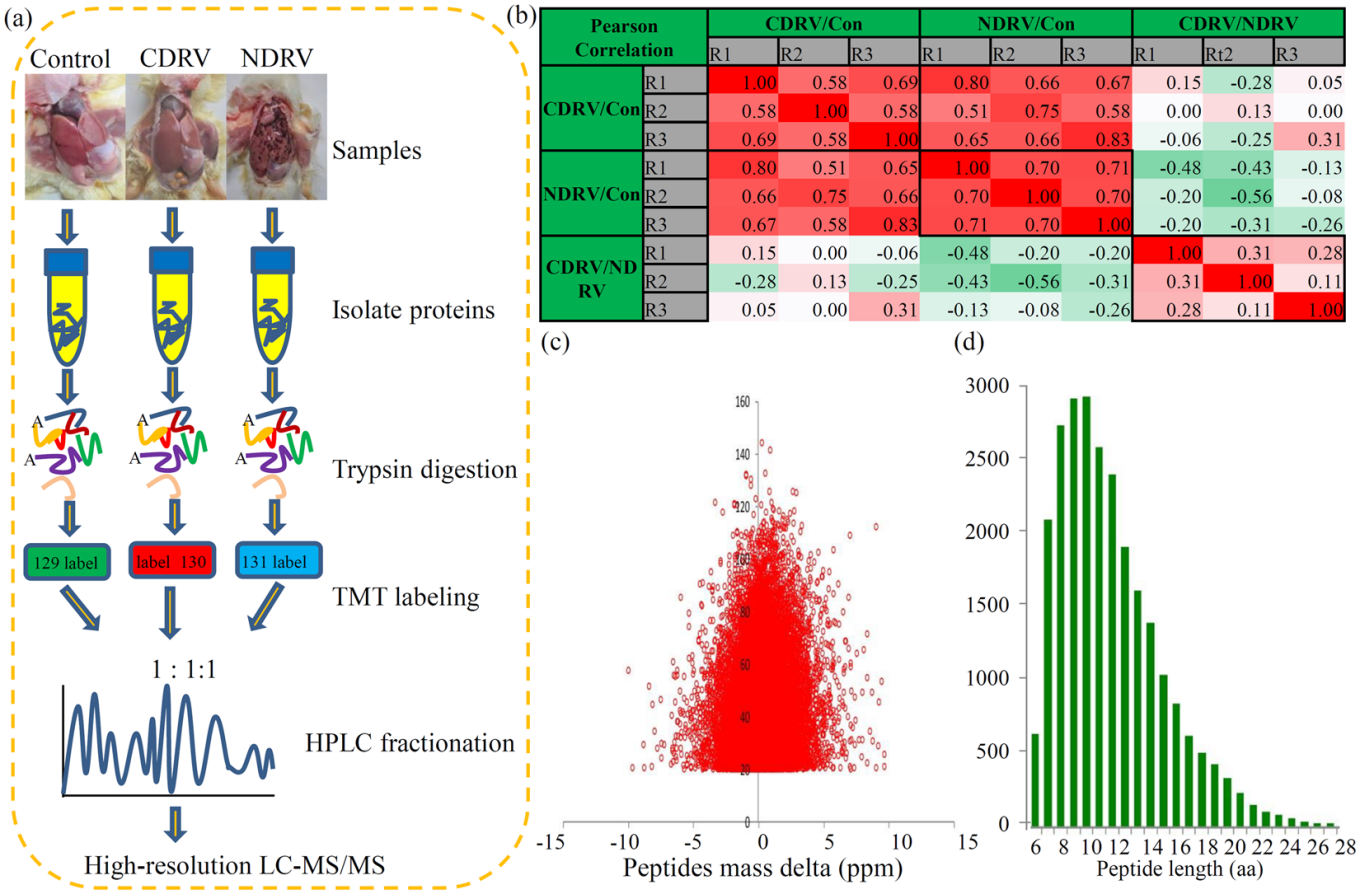
Experimental strategy for quantitative proteome analysis and QC validation of MS data. (**a**) Proteins were extracted in three biological replicates for each sample group. Proteins were trypsin digested and analyzed by HPLC-MS/MS. (**b**) Pearson’s correlation of protein quantitation. (**c**) Mass delta of all identified peptides. (**d**) Length distribution of all identified peptides.

To quantify the dynamic shifts in the whole proteomes of different liver samples (three repeats × three groups), an integrated proteomic approach was carried out. Basic experiment flow was showed in Fig. 1a. Pair-wise Pearson’s correlation coefficients of the nine sample groups showed a great repeatability of our study (Fig. 1b). The MS data produced a large number of peptides with mass errors < 0.02 Da, suggesting a fine mass accuracy of our MS data (Fig. 1c). Moreover, most peptides contained 8 to 17 amino-acid residues, indicating that the sample preparation reached the general standards (Fig. 1d).

### Quantitative proteomic analysis revealed a number of DEPs

**Figure 2:**
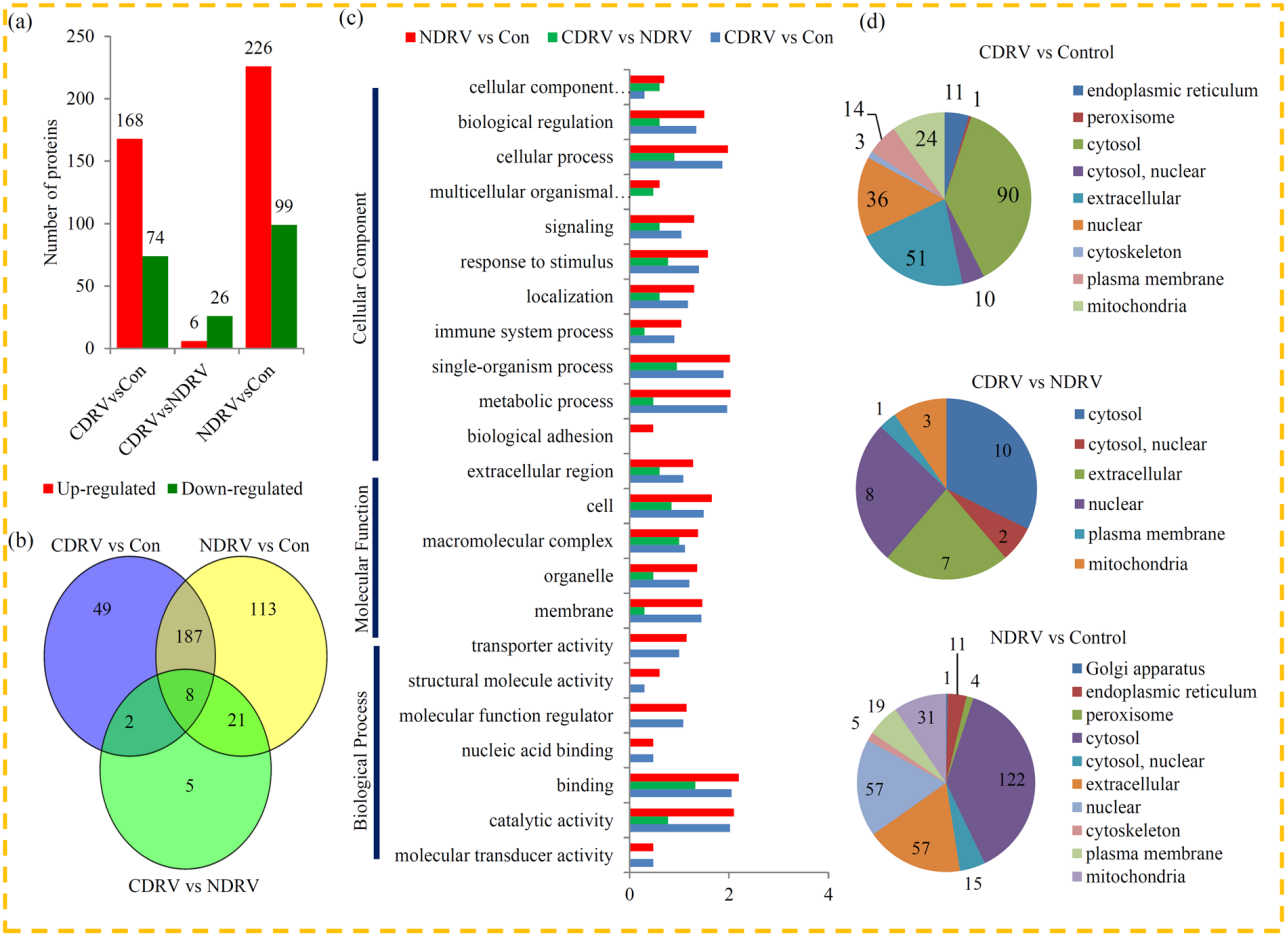
Classification and annotation of the DEPs. (**a**) The numbers of the up- and down-regulated proteins in each comparison. (**b**) Venn diagram showed the numbers of DEPs in different comparisons. (**c**) GO analysis of the DEPs in different comparisons. All proteins were classified by GO terms based on their cellular component, molecular function, and biological process. (**d**) Subcellular locations of the DEPs in different comparisons.

In our study, a total of 5571 proteins were identified, among which 5015 proteins were quantified. The basic information of the identified proteins, including annotations, functional classifications, functional enrichments, and subcellular localizations was listed in Table S1. In the CDRV vs control comparison, 242 proteins, including 168 up- and 74 down-regulated proteins, were identified. In the NDRV vs control comparison, 325 proteins, including 226 up- and 99 down-regulated proteins, were identified (Fig. 2a). The differences in the proteins between CDRV and NDRV infections were also compared, pointing out only 6 up- and 26 down-regulated proteins. A venn diagram displayed that a large number of DEPs (187 proteins) were commonly changed by both the CDRV and NDRV infections (Fig. 2b).

Under the CDRV infection, the greatest up-regulated proteins (over 5 folds) were serum amyloid protein (U3IC83) and alpha-1-acid glycoprotein (U3I466); and the greatest down-regulated proteins were malic enzyme (U3IL38), fatty acid synthase (R0KBE2), and acetoacetyl-CoA synthetase (U3IQX8) **(**Table S2**)**. Under the NDRV infection, serum amyloid protein (U3IC83), alpha-1-acid glycoprotein (U3I466), interferon alpha-inducible protein 6 (R0JE94), and histone H5 were induced over 5 folds; and stearoyl-CoA desaturase (A0A0H3U2H5), fatty acid synthase (R0KBE2) and malic enzyme (U3IL38) were largely reduced (Table S3).

All identified proteins and the DEPs were classified into different GO terms representing three categories: ‘cellular component’, ‘biological process’ and ‘molecular function’. In detail, 49.8% (2772 proteins), 30.4% (1694 proteins) and 28.9% (1611 proteins) of the identified proteins were annotated as ‘binding’, ‘catalytic activity’ and ‘cellular process’, respectively. In the CDRV vs control comparison, 46.7% (113 proteins), 43.4% (105 proteins) and 38.0% (92 proteins) of the DEPs were classified into the ‘binding’, ‘catalytic activity’ and ‘metabolic process’ GO terms, respectively. In the NDRV vs control comparison, a similar GO distribution pattern was observed (Fig. 2c). In the CDRV vs NDRV comparison, 65.6% (21 proteins), 31.2% (10 proteins) and 28.1% (9 proteins) of the DEPs were classified into the ‘binding’, ‘macromolecular complex’ and ‘single-organism process’ GO terms, respectively. Furthermore, the DEPs were also grouped into different subcellular location terms (Fig. 2d).

In total, only 32 DEPs, including 6 CDRV infection preferentially accumulated and 26 NDRV infection preferentially accumulated proteins, were observed between the CDRV and NRDV infections (Table S4).

### Enrichment analysis of the DEPs under the C/NDRV infections

**Figure 3:**
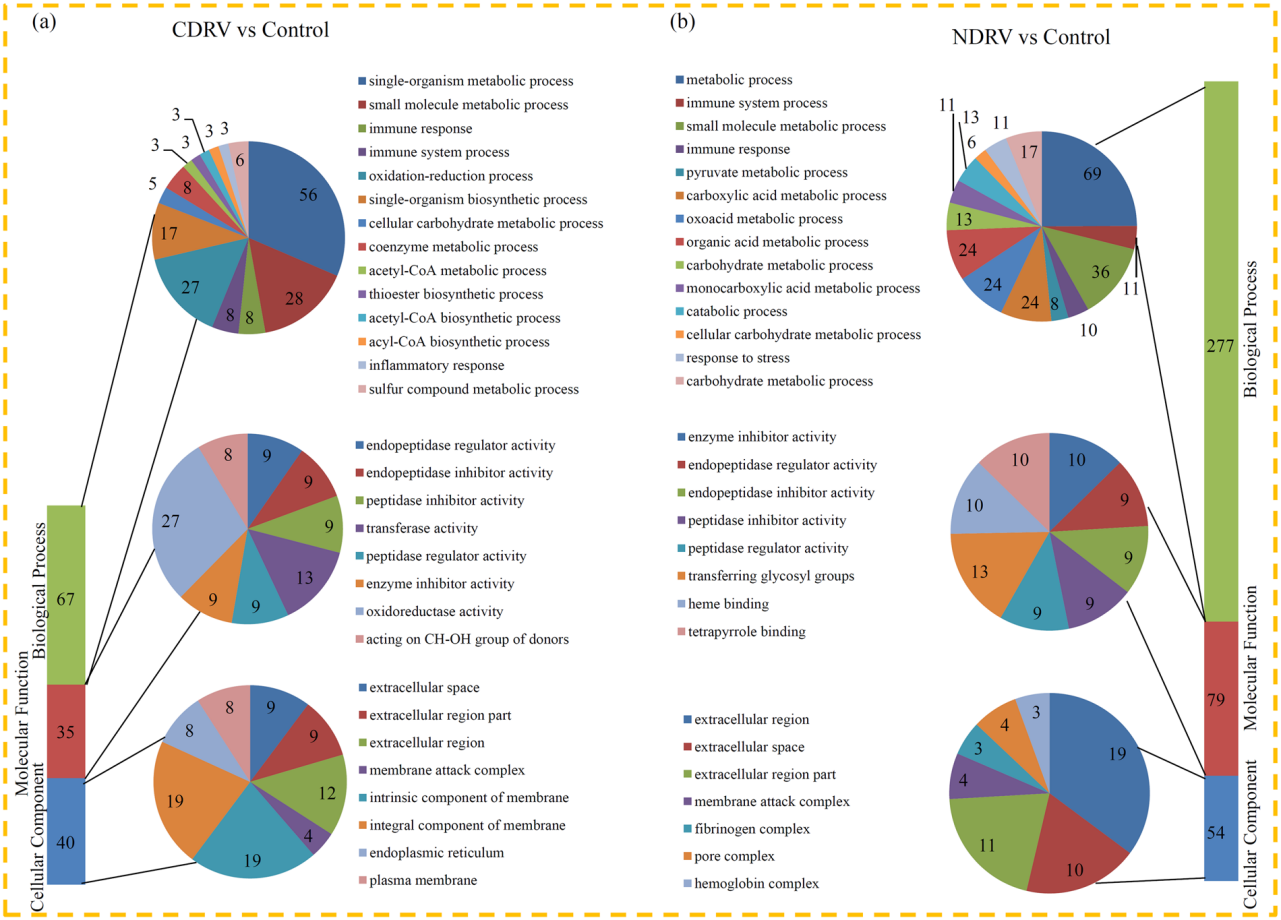
GO enrichment analysis of the DEPs. (**a**) Distribution of the DEPs in CDRV vs Control comparison with GO annotation. Different color blocks represent different terms, including cellular component, molecular function, and biological process. Number of DEPs in each second level term was showed in a pie chart. (**b**) Distribution of the DEPs in NDRV vs Control comparison with GO annotation. Different color blocks represent different terms, including cellular component, molecular function, and biological process. Number of DEPs in each second level term was showed in a pie chart.

Under C/NDRV infections, most DEPs were assigned to at least one GO term. In the CDRV vs control comparison, the significantly enriched ‘biological process’ GO terms were ‘single-organism metabolic process’ (56 DEPs), ‘small molecule metabolic process’ (28 DEPs) and ‘oxidation-reduction process’ (27 DEPs); the largest enriched GO terms associated with ‘molecular function’ were ‘oxidoreductase activity’ (27 DEPs); and the top three significantly enriched ‘cellular component’ GO terms were ‘integral component of membrane’ (19 DEPs), ‘intrinsic component membrane’ (19 DEPs) and ‘extracellular region’ (12 DEPs) (Fig. 3a). In the NDRV vs control comparison, the significantly enriched ‘biological Process’ GO terms were associated with ‘metabolic process’ (69 DEPs), ‘small molecule metabolic process’ (36 DEPs), ‘organic acid metabolic process’ (24 DEPs), ‘carboxylic acid metabolic process’ (24 DEPs) and ‘oxo-acid metabolic process’ (24 DEPs); within the ‘molecular function’ category, the most represented GO terms were ‘transferring glycosyl groups’ (13 DEPs), ‘enzyme inhibitor activity’ (10 DEPs), ‘heme binding’ (10 DEPs), and ‘tetrapyrrole binding’ (10 DEPs); and the most enriched GO terms within the ‘molecular function’ category were ‘extracellular region’ (19 DEPs), ‘extracellular region part’ (11 DEPs) and ‘extracellular space’ (10 DEPs) (Fig. 3b).

**Figure 4:**
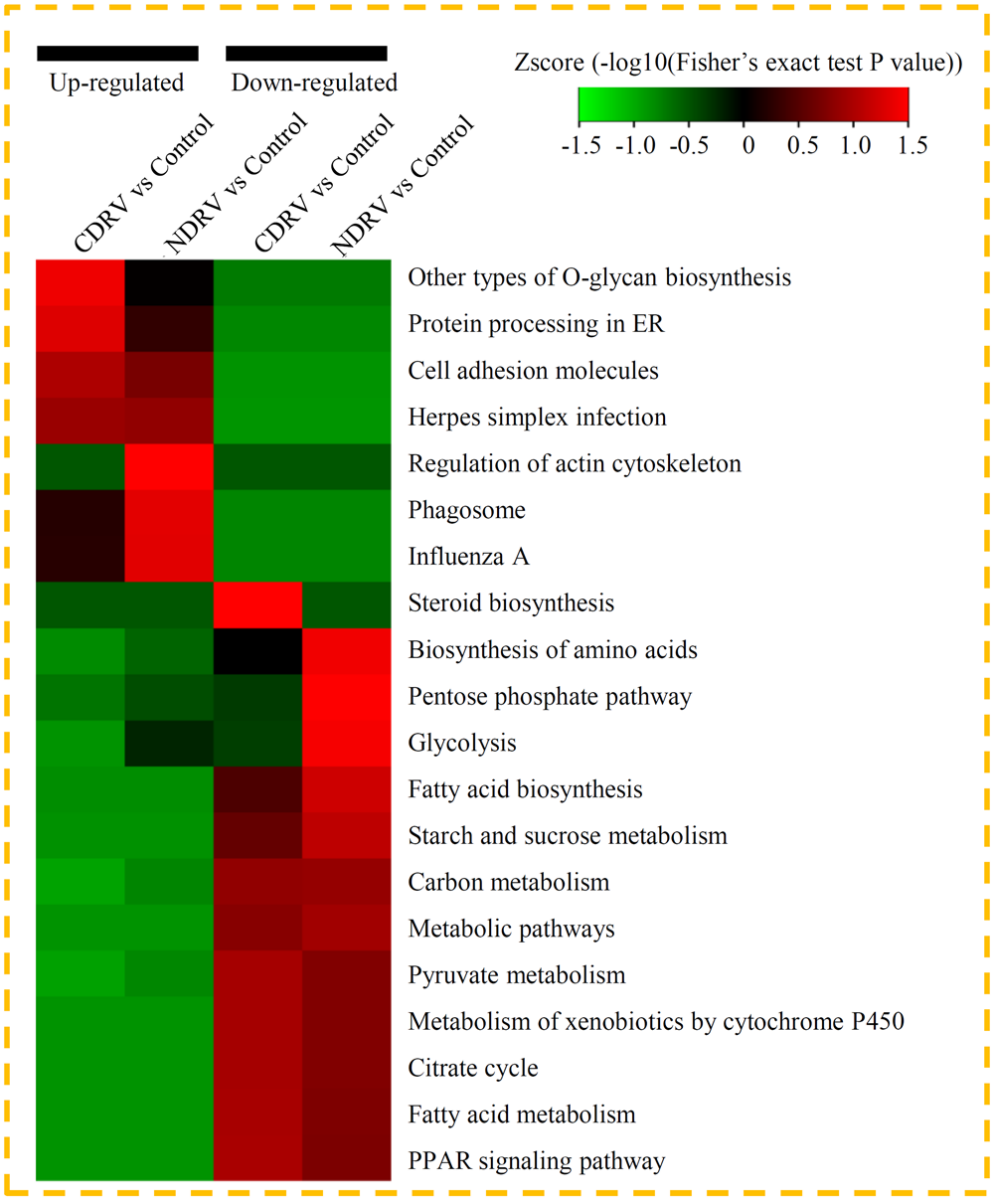
KEGG and protein domain enrichment analysis of the DEPs. (**a**) Significantly enriched KEGG terms of the up- and down-regulated DEPs in different comparisons. (**b**) Significantly enriched protein domains of the DEPs.

Under the CDRV infection, 107 DEPs were grouped into different KEGG pathways, of which eight metabolic pathways were enriched (*P* < 0.05). Most of the CDRV infection induced proteins were enriched in four metabolic pathways, including the ‘Other types of O-glycan biosynthesis’, ‘Protein processing in ER’, ‘Cell adhesion molecules’, and ‘Herpes simplex infection’ pathways. Addition to above four pathways, the NDRV infection induced protein were also enriched in another three pathways, such as the ‘Regulation of actin cytoskeleton’, ‘Phagosome’ and ‘Influenza A’ pathways. The proteins that were down-regulated by both of the CDRV and NDRV infections were enriched in the ‘Fatty acid biosynthesis’, ‘Starch and sucrose metabolism’, ‘Carbon metabolism’, ‘Metabolic pathways’, ‘Pyruvate metabolism’, ‘Metabolism of xenobiotics by cytochrome P450’, ‘Citrate cycle’, ‘Fatty acid metabolism’ and ‘PPAR signaling pathway’ pathways (Fig. 4).

Under the CDRV infection, the most significantly enriched domains in the DEPs were ‘Rossmann-like’, ‘Biotin’ and ‘single hybrid motif’ domains (Fig. S1a). Under the NDRV infection, the most significantly enriched domains in the DEPs were ‘Serine proteases, trypsin domain’, ‘Fibrinogen, alpha/beta/gamma chain, coiled coil domain’ and ‘AMP-dependent synthetase/ligase’ domains (Fig. S1b).

### PPI networks for the DEPs

**Figure 5:**
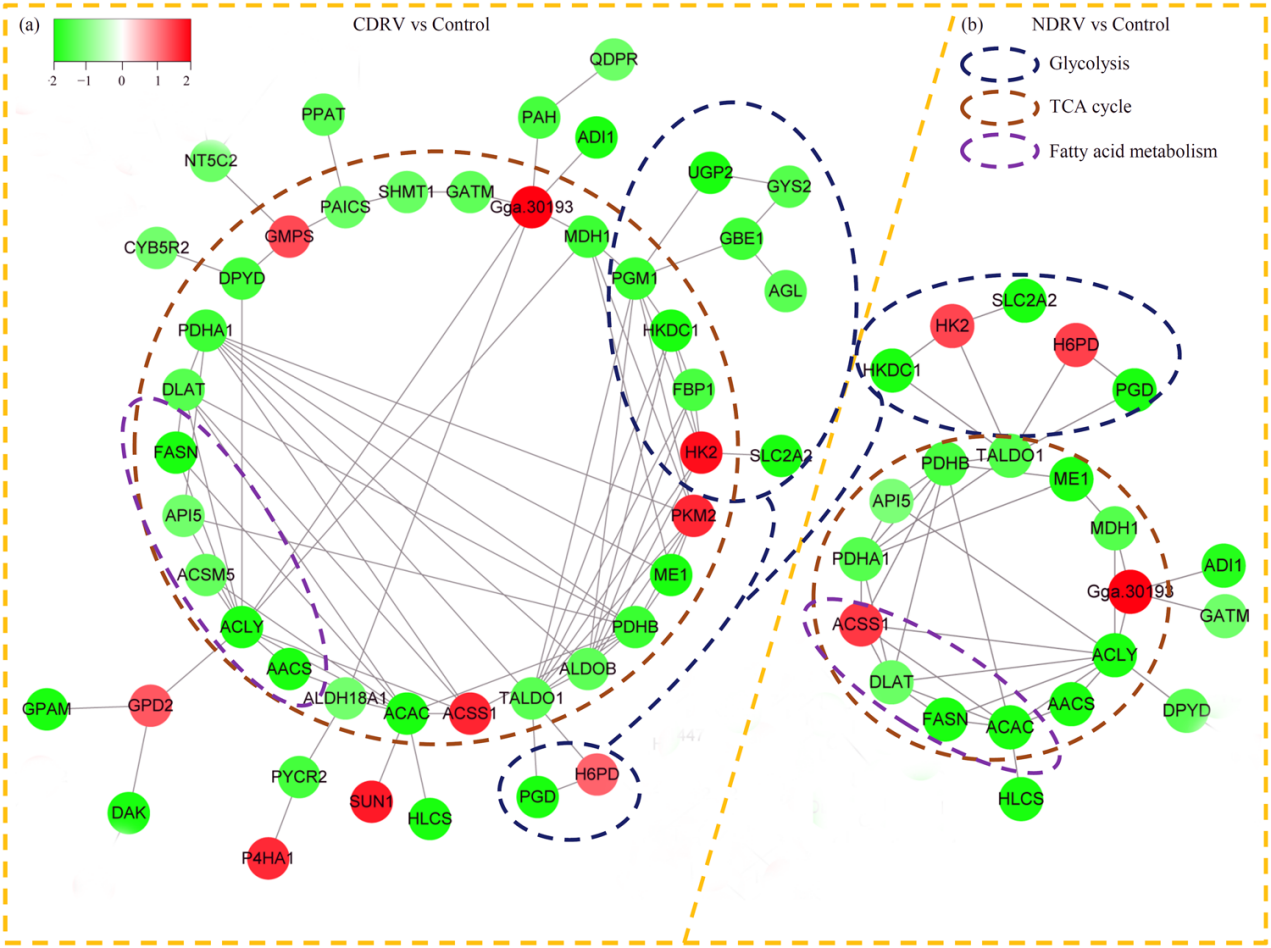
Protein interaction networks of the DEPs. The PPI networks for the DEPs in the CDRV vs Control and NDRV vs Control comparisons were analyzed using Cytoscape software version 3.6.1 (http://cytoscape.org/). Cycles indicated enriched interaction clusters. Blue cycle indicated the DEPs related to Glycolysis; brown cycle indicated the DEPs related to TCA cycle; and purple cycle indicated the DEPs related to Fatty acid metabolism.

To predict the biological functions of newly identified proteins, PPI networks were generated to reveal the relationship between the DEPs. In our study, the PPIs of the DEPs under both of the C/NDRV infections were analyzed to comprehend the dynamic shifts in metabolic pathways. In total, 46 DEPs in the CDRV vs control comparison and 22 DEPs in the NDRV vs control comparison were treated as network nodes. Then, three highly enriched interaction clusters, including glycolysis, TCA cycle and fatty acid metabolism, were identified in the networks (Fig. 5).

### DEPs related to various key metabolic pathways

**Figure 6:**
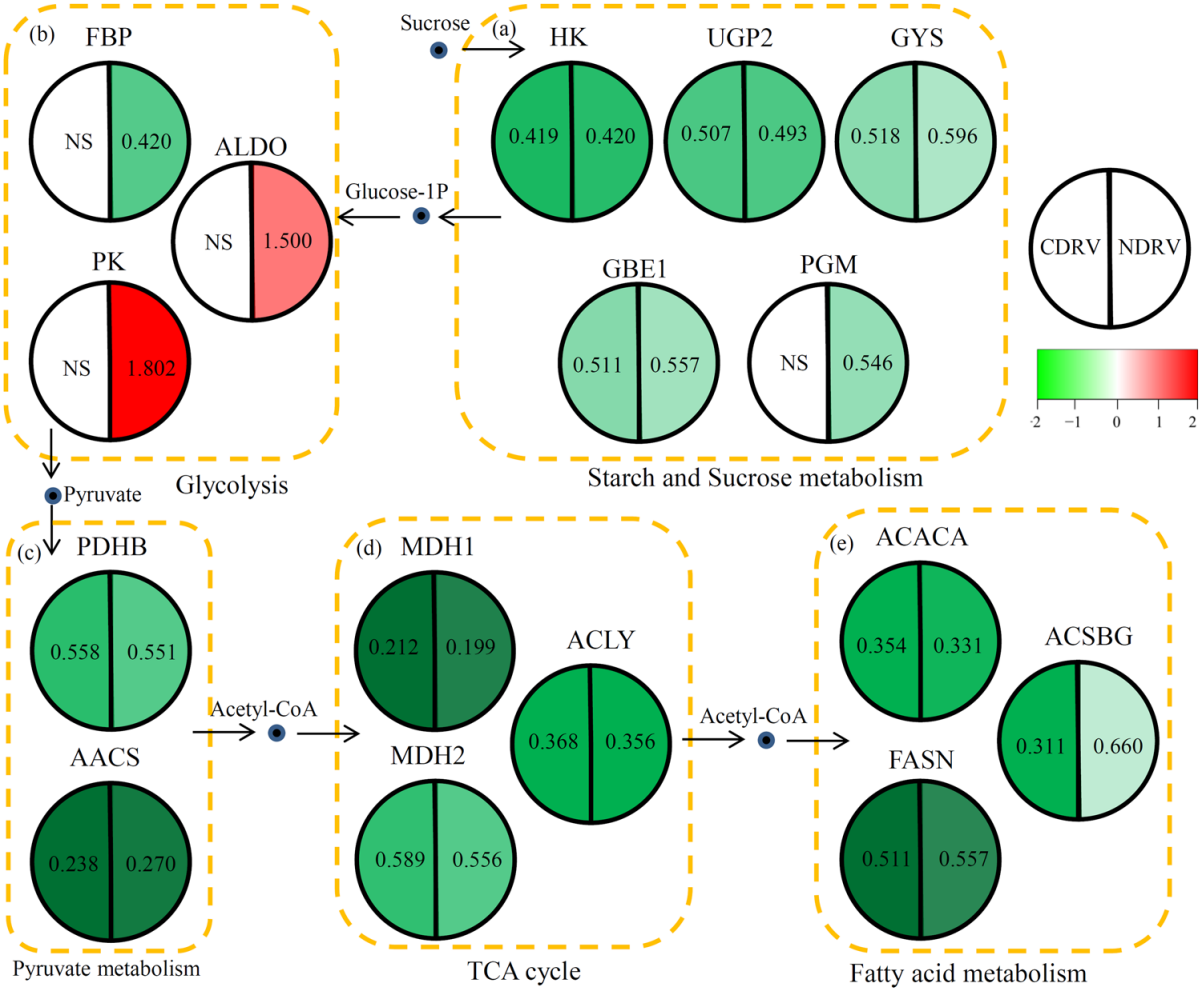
Involvement of basal and secondary metabolisms in responses to classical/novel duck reovirus infections. Schematic representation of the proteins involved in basal and secondary metabolisms, including starch and sucrose metabolism (**a**), glycolysis (**b**), pyruvate metabolism (**c**), TCA cycle (**d**) and fatty acid metabolism (**e**). HK: hexokinase [EC:2.7.1.1]; UGP2: UTP–glucose-1-phosphate uridylyltransferase [EC:2.7.7.9]; GYS: glycogen synthase [EC:2.4.1.11]; GBE1: 1,4-alpha-glucan branching enzyme [EC:2.4.1.18]; PGM: phosphoglucomutase [EC:5.4.2.2]; FBP: fructose-1,6-bisphosphatase I [EC:3.1.3.11]; ALDO: fructose-bisphosphate aldolase, class I [EC:4.1.2.13]; PK: pyruvate kinase [EC:2.7.1.40]; PDHB: pyruvate dehydrogenase E1 component beta subunit [EC:1.2.4.1]; AACS: acetoacetyl-CoA synthetase [EC:6.2.1.16]; MDH1: malate dehydrogenase [EC:1.1.1.37]; MDH2: malate dehydrogenase (NADP+) [EC:1.1.1.40]; ACLY: ATP citrate (pro-S)-lyase [EC:2.3.3.8]; ACACA: acetyl-CoA carboxylase [EC:6.4.1.2]; FASN: fatty acid synthase, animal type [EC:2.3.1.85]; and ACSBG: long-chain-fatty-acid–CoA ligase ACSBG [EC:6.2.1.3].

KEGG analysis indicated that various key metabolic pathways, from starch and sucrose metabolism to fatty acid metabolism, involved in the responses to the C/NDRV infections (Table S5 and S6). For starch and sucrose metabolism, four enzymes (HK, UGP2, GYS and GBE1) were inhibited by both of the CDRV and NDRV infections and one enzyme (PGM) was only reduced by the NDRV infection. For glycolysis pathway, no significantly changes were detected under the CDRV infection, and FBP was down-regulated and two enzymes (PK and ALDO) were up-regulated under the NDRV infection. Besides, two pyruvate metabolism-related enzymes (PDHB and AACS), three TCA cycle associated enzymes (MDH1, MDH2 and ACLY) and three fatty acid metabolism-related enzymes (ACACA, FASN and ACSBG), were down-regulated under both of the CDRV and NDRV infections (Fig. 6).

### DEPs involved in the protease systems

The complement system and the coagulation system, main columns of innate immunity and homeostasis, have fundamental clinical implications in the context of diseases with inflammatory pathogenesis^21^. In our study, a number of DEPs involved in the protease systems were identified. For the coagulation system, six coagulation factors, including coagulation II, V, VII, IX, X and XIII, were identified, among which five were quantified, except for coagulation VII. Coagulation factor XIII (R0KCX2) was significantly up-regulated under both of the CDRV and NDRV infections. Besides, coagulation factors V (U3IF51) and X (U3I5A6) were significantly induced by the NDRV infection. For the fibrinolytic system, three fibrinogens, including alpha (R0JSX9), beta (R0JS80) and gamma (U3IA23), were identified. Interestingly, all three fibrinogens were significantly up-regulated by both of the CDRV and NDRV infections. For the complement system, 16 complement factors were identified, among which 12 complement factors were significantly induced by the CDRV infection and 13 complement factors were significantly induced by the NDRV infection (Table 1).

**Table 1:** The detail information of proteins involved in serine protease systems.

Protein accession	Protein description	MW [kDa]	calc.pI	CDRV/Con Ratio	CDRV/Con P value	NDRV/Con Ratio	NDRV/Con P value
Coagulation System							
U3J210	Coagulation factor II	69.154	5.91	1.077	2.37E-01	1.416	6.34E-03
U3IF51	Coagulation factor V	241.431	7.08	1.186	4.52E-01	2.018	1.25E-02
U3I7N5	Coagulation factor VII	44.957	5.77	none	none	none	none
R0JLH6	Coagulation factor IX	29.67	5.16	1.073	4.43E-01	1.001	9.92E-01
U3I5A6	Coagulation factor X	54.019	5.55	1.177	2.45E-01	1.665	1.12E-02
R0KCX2	Coagulation factor XIII A chain	83.716	6.09	2.127	6.66E-03	2.298	6.43E-03
Fibrinolytic System							
R0JSX9	Fibrinogen alpha chain	82.382	6.09	1.545	1.71E-02	4.182	3.95E-02
R0JS80	Fibrinogen beta chain	51.826	7.75	1.748	1.66E-02	4.367	1.11E-03
U3IA23	Fibrinogen gamma chain	49.967	5.66	1.486	3.46E-02	3.493	1.26E-03
Complement System							
R0LGG0	Complement C1q subcomponent subunit A	24.927	8.72	2.293	2.70E-04	2.087	4.12E-03
R0LW73	Complement C1q subcomponent subunit B	31.918	9.23	2.458	2.12E-04	2.125	2.04E-03
U3IES4	Complement C1r subcomponent	79.899	6.87	1.693	6.23E-02	1.554	2.10E-02
U3ID07	Complement C1s subcomponent	77.741	5.07	1.959	1.43E-03	1.946	9.14E-03
U3I8R2	Complement C4	188.759	6.6	1.719	1.58E-02	2.080	7.98E-03
U3IIY3	Complement C5	173.179	6.89	1.647	1.26E-04	1.749	1.71E-03
U3IJ87	Complement C5	15.588	8.1	1.615	1.88E-01	1.658	1.85E-01
B5AG23	Complement component 3d	36.671	8.81	1.573	7.76E-01	1.952	6.94E-01
U3IQV8	Complement component C6	105.688	7.2	1.258	3.47E-02	1.268	2.78E-02
U3INH5	Complement component C7	94.339	5.68	1.826	6.50E-05	3.321	8.83E-05
U3IKH3	Complement component C8 alpha chain	60.306	5.99	2.018	1.29E-02	2.526	2.58E-03
U3IKF3	Complement component C8 beta chain	64.231	7.93	1.771	1.10E-02	1.999	3.52E-03
U3IN55	Complement component C9	66.437	5.43	1.823	1.43E-03	2.100	2.76E-03
U3IUA7	Complement factor H	135.721	7.02	1.812	6.05E-04	1.613	7.77E-03
R0KKF7	Complement factor H-related protein 3	7.335	7.74	1.676	6.04E-04	1.975	5.77E-03
U3ITG0	Complement factor I	53.864	7.36	1.004	9.67E-01	1.151	2.85E-01

### Identification of differential expressed molecular chaperones

The importance of molecular chaperones in liver immunity against various viruses has been well-studied^22,23^. In our study, a large number of molecular chaperones, including the members of Hsp family, the members of BAG family and others, were identified. For the Hsp family, ‘DnaJ subfamily B member 11’ (U3IC97) and DnaJ subfamily B member 1’ (U3IQM1) were siginificantly up-regulated by both of the CDRV and NDRV infections. For the BAG family, only ‘BAG family molecular chaperone regulator 5’ (U3I2D5) was significantly induced by the NDRV infection. For others, no significant changes were observed (Table 2).

**Table 2:** The detail information of molecular chaperone.

Protein accession	Protein description	MW [kDa]	calc.pI	CDRV/Con Ratio	CDRV/Con P value	NDRV/Con Ratio	NDRV/Con P value
Hsp family							
R0L7G7	Calcyclin-binding protein	24.926	8.72	1.085	3.62E-01	1.002	9.91E-01
U3HZP6	Cysteine and histidine-rich domain-containing protein 1	37.37	7.8	1.006	8.67E-01	0.970	5.91E-01
U3IQM1	DnaJ subfamily B member 1	18.363	7.39	1.151	1.54E-01	1.325	1.41E-02
U3IC97	DnaJ subfamily B member 11	40.519	6.87	1.520	3.82E-04	1.444	1.09E-03
R0LC09	DnaJ-like protein subfamily A member 1	33.149	6.79	0.935	1.87E-01	1.007	8.66E-01
R0KXH1	DnaJ-like protein subfamily A member 2	42.841	6.61	0.986	7.33E-01	0.956	2.74E-01
U3J3I0	DnaJ-like protein subfamily A member 3	43.156	8.85	1.093	1.29E-01	1.165	3.65E-02
U3I1F6	DnaJ-like protein subfamily A member 4	39.702	5.78	1.282	6.11E-03	1.412	1.73E-03
U3IE39	DnaJ-like protein subfamily B member 4	38.707	9.01	1.093	2.69E-01	1.314	2.18E-02
R0JSM9	Heat shock protein HSP 90-alpha	84.508	5.08	1.049	2.99E-01	1.027	6.54E-01
R0L654	NudC domain-containing protein 1	61.722	5.17	0.968	6.55E-01	0.977	7.31E-01
U3J0V5	NudC domain-containing protein 2	11.129	4.88	1.662	7.72E-01	1.731	7.57E-01
U3IPX3	NudC domain-containing protein 3	10.72	8.16	1.052	1.29E-01	0.795	3.99E-02
U3INS4	Prostaglandin E synthase 3	18.651	4.54	1.158	6.05E-03	1.050	3.26E-01
U3IP36	Stress-70 protein	73.683	5.96	0.948	2.01E-01	0.927	2.90E-02
U3IYB8	Hsp90 co-chaperone Cdc37	27.833	6.21	1.148	2.72E-02	1.020	7.36E-01
BAG family							
R0JKB8	BAG family molecular chaperone regulator 1	21.79	6.28	1.114	8.86E-01	1.152	8.52E-01
U3IP81	BAG family molecular chaperone regulator 3	53.931	6.55	0.942	4.90E-01	1.275	8.29E-02
U3I2D5	BAG family molecular chaperone regulator 5	51.23	6.15	1.334	4.41E-02	1.575	2.36E-02
Others							
U3J4H9	Chaperone activity of bc1 complex-like	72.747	7.08	1.223	2.72E-02	1.269	4.41E-03
U3IUA1	Cytochrome C oxidase copper chaperone	5.852	8.02	1.283	1.37E-01	1.115	3.17E-01
U3J0C5	GrpE protein homolog	24.939	8.59	1.058	4.66E-01	0.972	6.53E-01
U3ITZ9	LDLR chaperone MESD	25.34	8.15	1.514	3.04E-02	1.227	5.79E-02
U3IC16	Mitochondrial chaperone BCS1	47.039	8.78	0.946	4.19E-01	0.855	1.64E-01
U3IPW0	Mitochondrial import inner membrane translocase subunit TIM44	51.539	8.12	1.133	1.98E-02	1.033	3.67E-01
U3J5M7	Proteasome assembly chaperone 1	23.716	5.36	0.946	2.92E-01	0.888	1.10E-01
U3IRF8	Proteasome assembly chaperone 2	29.737	7.17	1.012	7.97E-01	0.960	4.94E-01
U3ILR9	Torsin-1A	32.586	7.52	1.259	7.64E-01	1.310	7.27E-01
U3II70	Tubulin-specific chaperone A	11.17	4.93	0.928	2.07E-01	0.872	6.51E-02
R0JMP4	Tubulin-specific chaperone cofactor E-like protein	48.272	5.45	1.186	7.01E-02	1.318	4.65E-02
U3ITR5	Tubulin-specific chaperone D	128.223	6.89	0.978	7.94E-01	0.862	2.02E-01
U3IDV7	Tubulin-specific chaperone E	57.735	7.15	1.184	9.16E-03	1.107	1.49E-01
U3IIV4	Ubiquinol-cytochrome C reductase complex chaperone CBP3-like protein	34.786	7.97	1.010	8.45E-01	0.981	7.79E-01

### Verification of the changes in the proteins responsive to the C/NDRV infections

**Figure 7:**
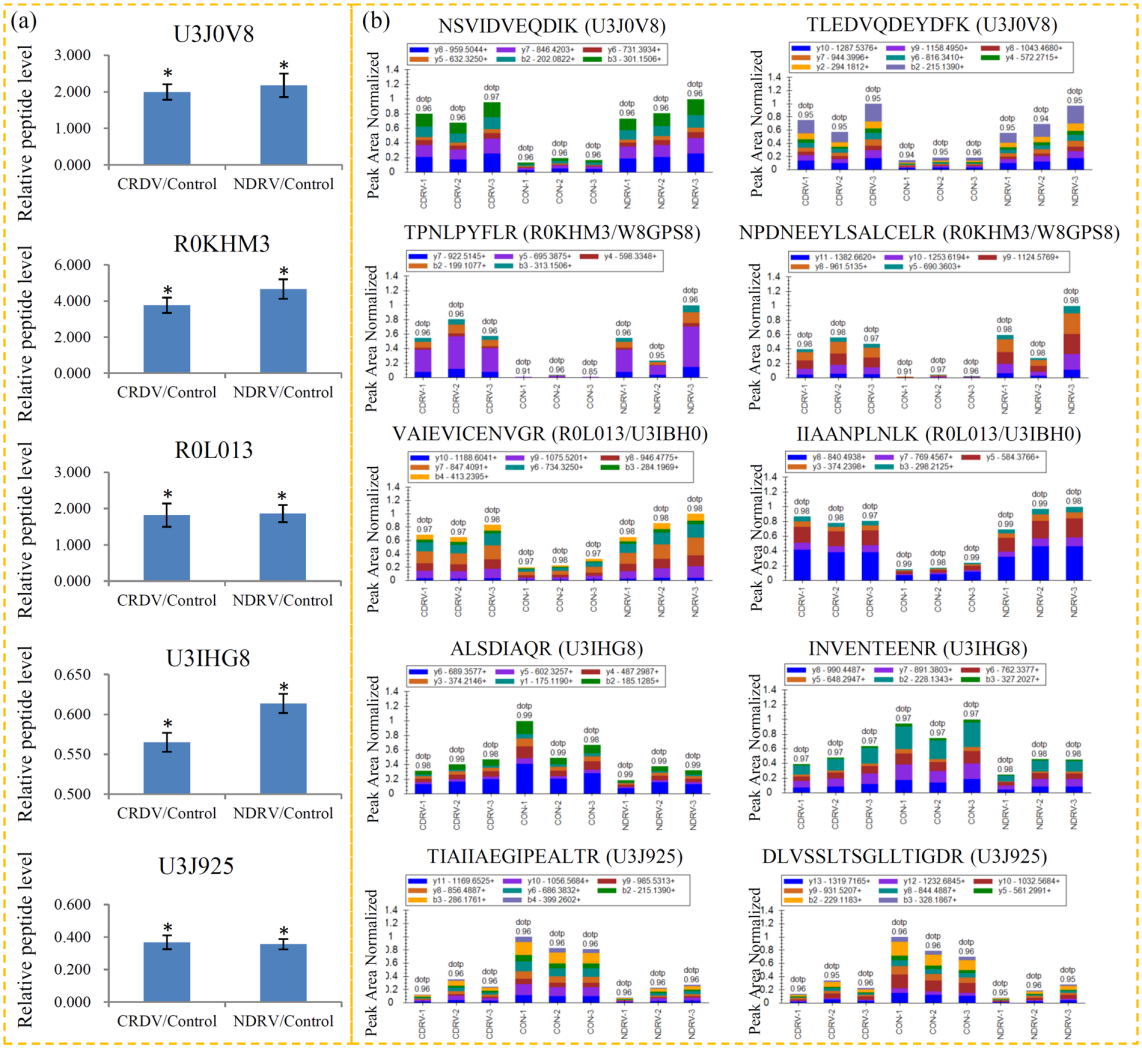
Verification of the changes in the proteins respond to C/NDRV infections using PRM. Five representative proteins randomly selected for TMT-LC-MS/MS (**a**) and PRM (**b**) verification. For each protein, the abundances of two peptides were determined.

Because virus response is extremely complex, it is difficult to identify a single sensitive biomarker. Identification of a panel of C/NDRV infection responsive proteins would be feasible to offer better understanding to this complex biological process. PRM was applied to validate the differential expression of several key proteins involved in the responses to the C/NDRV infections. In total, 14 key proteins involved in various biological processes were selected for the PRM verification (Table S7). The relative abundances of several key proteins from different sample groups were presented in Fig. 7. The trend of these DEPs checked by PRM was agreed with the results of the TMT-label quantification.

## Discussion

C/NDRV mainly infect ducklings resulting to general weakness, diarrhea, serofibrinous pericarditis and swollen liver covered with small white necrotic foci^13^. As fatal pathogenic viruses, C/NDRV have caused huge economic losses of the duck industry over the past several decades^24^. The liver, one of the most important organs, plays vital roles in digestion, metabolism process and innate immune system response in ducks^25^. However, little information of the protein profile of the liver cells infected with C/NDRV in ducks was available. Here, an integrated approach involving TMT labeling and LC-MS/MS was applied to identify the DEPs in liver cells of *C*. *moschata* under the C/NDRV infections.

Recently, several proteomic analyses have provided a number of responsive proteins involved in various virus infections. For example, 19 and 175 differentially accumulated cellular proteins were identified in *tembusu* virus-infected BHK-21 duck cells at 24 and 48 hpi, respectively^26^. In primary duck hepatocytes, 75 DEP spots under *duck hepatitis B* virus infection were revealed by 2-D analysis^27^. Quantitative proteomic analysis of tembusu virus infected duck ovarian follicles identified 131 DEPs^17^. In our study, 5571 proteins were identified, including 242 and 325 DEPs under the CDRV and NDRV infections, respectively. The number of DEPs identified in our study was larger than that in the previous studies, suggesting a deeper exploration of new proteins that were potentially involved, directly and indirectly, in the immune responses to the C/NDRV infections.

CDRV was firstly described in South Africa in 1950 and isolated in France in 1972^6,28^. Since 2000, a NDRV-related disease was found in the major duck feeding regions of China^7^. The differences in genome segments between CDRV and NRDV have been completely determined^11–13,29^. However, information on the differential host responses to the CDRV and NRDV infections is limited. In our study, only 32 DEPs were identified between CDRV and NDRV infections, pointing out an unexpectedly similarity in the host responsive patterns between CDRV and NRDV infections (Fig. 2a). From CDRV to NDRV, along with the evolution of the virus, the pathogenicity of NDRV was greatly differ from that of CDRV^30^. These 32 significantly DEPs may provide an explanation of the differences in the pathogenicity between CDRV and NDRV (Table S4). Interestingly, three fibrinogens (alpha, beta and gamma), were siginificantly high accumulated in the NDRV infected liver cells compared to the CDRV infected liver cells, giving a lot of large blood spots in the NDRV infected liver.

A general assessment of metabolic regulation under virus infections has been widely discussed^31–33^. KEGG enrichment analysis revealed that various metabolic pathways were significantly inhibited by the C/NDRV infections. Neutral fat accumulation in liver cells is caused by the infections of pathogenic microorganisms^34^. Viral infection can result to an abnormality of fatty acid metabolism in livers^35^. A previous study indicated that the contents of free fatty acids were significantly induced by the MDRV infection in liver. Moreover, the expression of several fatty acid metabolism associated enzymes were significantly inhibited in the livers^16^. In our study, a number of fatty acid metabolism-related enzymes were identified as down-regulated proteins under both of the CDRV and NDRV infections. Thus, C/NDRV infections may reduce the decomposition of fatty acids and cause fat accumulation in the infected livers.

The network of serine proteases play a central role in the innate immune reactions, such as removing of pathogens and repairing of damaged tissues. It is well known that the serine protease systems are consisted of several autonomous proteolytic cascade systems, such as the complement, coagulation and fibrinolytic systems^21,36^. Activated coagulation cascade can prevent the invasion of microorganisms and the subsequent inflammatory responses^37^. In our study, a total of six coagulation factors were identified, among which coagulation factor XIII was significantly up-regulated by both of the CDRV and NDRV infections. The traditional functions of coagulation factor XIII in the stabilization of fibrin clot and protection of fibrin against fibrinolysis have been well summarized in the last decade^38^. Our data suggested an involvement of coagulation factor XIII in fibrinolysis and thrombosis under the C/NDRV infections. Fibrin, the ultimate substrate for fibrinolysis, plays an essential role in the hemostasis^39^. Fibrinogen, a soluble 340 kDa protein with three distinct disulfide-linked polypeptide chains, including α, β and γ chains, is the major target of thrombin^40^. In our study, all the three chains of fibrinogen were significantly up-regulated by both of the CDRV and NDRV infections. Higher concentrations of fibrinogen under the C/NDRV infections may enhance the control of blood coagulation^39^. Complement is composed of about 30 enzymes and plays roles in the defense against infections^37^. Under the C/NDRV infections, a number of complement enzymes were up-regulated, suggesting an activation of the serine protease systems in *C*. *moschata*.

Chaperones are important small compounds to maintain the stability and folding of unfolded or misfolded proteins^41^. A central role of chaperones in the immune networks has been well elucidated^42^. Hsp family members, more generally named as ‘stress proteins’, are involved in the ancient defense system of living organisms^43^. For example, a positive cellular role of Hsp90 during the rotavirus infection has been reported^22^. In our study, a large number of molecular chaperones were identified, including 16 Hsp family members, three BAG family and 14 other chaperones. Interestingly, no significantly changes in the abundances of most identified molecular chaperones were observed. Therefore, the roles of these molecular chaperones in *C*. *moschata* under the C/NDRV infections need to be addressed in the future.

In summary, a large number of the classical/novel duck reovirus infection responsive proteins in *C*. *moschata* were identified by high throughput proteomics analysis. The DEPs between the control and infected cells showed various biological functions. The results will provide useful information about the pathogenicity of C/NDRV in ducks and new insights into the further study of the disease.

## Electronic supplementary material

Supplementary Information

Supplementary Dataset File

## References

[1] Malkinson M, Perk K, Weisman Y (1981). Reovirus infection of young Muscovy ducks (Cairina moschata). Avian Pathol.

[2] Pascucci *et al*. Evaluation of respiratory patterns of infants in the perioperative period. *Anesthesiology* **61** (1984).

[3] Heffels-Redmann U, Muller H, Kaleta EF (1992). Structural and biological characteristics of reoviruses isolated from Muscovy ducks (Cairina moschata). Avian Pathol.

[4] Fuzhou. Discovery of the Pathogen of Muscovy Duck Liver White Spots Disease. Fujian Journal of Animal Husbandry & Veterinary, (2000).

[5] Liu Q (2011). Isolation and characterization of a reovirus causing spleen necrosis in Pekin ducklings. Vet Microbiol.

[6] Gaudry D, Charles JM, Tektoff J (1972). [A new disease expressing itself by a viral pericarditis in Barbary ducks]. C R Acad Sci Hebd Seances Acad Sci D.

[7] Yun T (2015). Development and application of an indirect ELISA for the detection of antibodies to novel duck reovirus. J Virol Methods.

[8] Wang D, Shi J, Yuan Y, Zheng L, Zhang D (2013). Complete sequence of a reovirus associated with necrotic focus formation in the liver and spleen of Muscovy ducklings. Vet Microbiol.

[9] Chen, S. *et al*. The Primary Study of Pathogen of Duck Hemorrhagic-necrotic Hepatitis. *Chinese Agricultural Science Bulletin*, (2009).

[10] Bi Z (2016). Induction of a robust immunity response against novel duck reovirus in ducklings using a subunit vaccine of sigma C protein. Sci Rep.

[11] Yun T (2013). Isolation and genomic characterization of a classical Muscovy duck reovirus isolated in Zhejiang, China. Infect Genet Evol.

[12] Wang S (2015). Sequence and phylogenetic analysis of M-class genome segments of novel duck reovirus NP03. Can J Vet Res.

[13] Yun T (2014). Genomic characteristics of a novel reovirus from Muscovy duckling in China. Vet Microbiol.

[14] Wang Q (2015). Spleen Transcriptome Profile of Muscovy Ducklings in Response to Infection With Muscovy Duck Reovirus. Avian Dis.

[15] Geng H (2009). Apoptosis induced by duck reovirusp10.8 protein in primary duck embryonated fibroblast and Vero E6 cells. Avian Dis.

[16] Wang, Q., Liu, M., Xu, L., Wu, Y. & Huang, Y. Transcriptome analysis reveals the molecular mechanism of hepatic fat metabolism disorder caused by Muscovy duck reovirus infection. *Avian Pathol*, 1–13 (2017). 10.1080/03079457.2017.1380294 28911249

[17] Han K (2016). Quantitative Proteomic Analysis of Duck Ovarian Follicles Infected with Duck Tembusu Virus by Label-Free LC-MS. Frontiers in Microbiology.

[18] Zeng T (2013). Comparative Proteomic Analysis of the Hepatic Response to Heat Stress in Muscovy and Pekin Ducks: Insight into Thermal Tolerance Related to Energy Metabolism. PLoS ONE.

[19] Huang, M. Q., Cheng, X. X., Chen, S. L., Zheng, M. & Chen, S. Y. Analysis of differentially expressed proteins in Muscovy duck embryo fibroblasts infected with virulent and attenuated Muscovy duck reovirus by two-dimensional polyacrylamide gel electrophoresis. *J Vet Med Sci*, (2017). 10.1292/jvms.17-0421 PMC5745192 29046506

[20] Thompson A (2003). Tandem mass tags: a novel quantification strategy for comparative analysis of complex protein mixtures by MS/MS. Anal Chem.

[21] Amara U (2008). Interaction Between the Coagulation and Complement System. Advances in Experimental Medicine & Biology.

[22] Dutta D (2009). The molecular chaperone heat shock protein-90 positively regulates rotavirus infectionx. Virology.

[23] Wang Y, Li J, Wang X, Sang M, Ho W (2013). Hepatic stellate cells, liver innate immunity, and hepatitis C virus. J Gastroenterol Hepatol.

[24] Zheng X (2016). A duck reovirus variant with a unique deletion in the sigma C gene exhibiting high pathogenicity in Pekin ducklings. Virus Res.

[25] Zheng A (2012). Proteomic analysis of liver development of lean Pekin duck (Anas platyrhynchos domestica). Journal of Proteomics.

[26] Sun, X. *et al*. Proteome analysis of Duck Tembusu virus (DTMUV)-infected BHK-21 cells. *Proteomics* **17** (2017). 10.1002/pmic.201700033 28516729

[27] Zhao Y (2010). Proteomic analysis of primary duck hepatocytes infected with duck hepatitis B virus. Proteome Science.

[28] Kaschula, V. R. A new virus disease of the muscovy duck (Cairina moschata Linn.) present in Natal. *Journal of the South African Veterinary Association*, (1950).

[29] Ma G (2012). Complete genomic sequence of a reovirus isolate from Pekin ducklings in China. J Virol.

[30] Chen Z, Zhu Y, Li C, Liu G (2012). Outbreak-associated Novel Duck Reovirus, China, 2011. Emerging Infectious Diseases.

[31] Elbacha T, Menezes MM, Mc AES, Solapenna M, Da PA (2004). Mayaro virus infection alters glucose metabolism in cultured cells through activation of the enzyme 6-phosphofructo 1-kinase. Molecular & Cellular Biochemistry.

[32] Kang ES, Galloway MS, Bean W, Cook GA, Olson G (1991). Acute alterations in the regulation of lipid metabolism after intravascular reexposure to a single bolus of homologous virus during influenza B infection in ferrets: possible model of epiphenomena associated with influenza. International Journal of Experimental Pathology.

[33] Fan W (2016). Metabolic product response profiles of Cherax quadricarinatus towards white spot syndrome virus infection. Developmental & Comparative Immunology.

[34] Liu Q (2017). Organochloride pesticides impaired mitochondrial function in hepatocytes and aggravated disorders of fatty acid metabolism. Scientific Reports.

[35] Magri MC (2017). Genetic variation in the microsomal triglyceride transfer protein (−493G/T) is associated with hepatic steatosis in patients infected with hepatitis C virus. Bmc Infectious Diseases.

[36] Adams MN (2011). Structure, function and pathophysiology of protease activated receptors. Pharmacology & Therapeutics.

[37] Markiewski MM, Nilsson B, Ekdahl KN, Mollnes TE, Lambris JD (2007). Complement and coagulation: strangers or partners in crime?. Trends in Immunology.

[38] Muszbek L, Bereczky Z, Bagoly Z, Komáromi I, Katona É (2011). Factor XIII: a coagulation factor with multiple plasmatic and cellular functions. Physiological Reviews.

[39] Chapin JC, Hajjar KA (2015). Fibrinolysis and the control of blood coagulation. Blood Reviews.

[40] Wolberg, A. S. Thrombin generation and fibrin clot structure. *Blood Reviews* **21**, 131–142. 10.1016/j.blre.2006.11.001 17208341

[41] Young JC, Agashe VR, Siegers K, Hartl FU (2004). Pathways of chaperone-mediated protein folding in the cytosol. Nature Reviews Molecular Cell Biology.

[42] Nardai G, Végh EM, Prohászka Z, Csermely P (2006). Chaperone-related immune dysfunction: an emergent property of distorted chaperone networks. Trends in Immunology.

[43] Zhao R (2005). Navigating the Chaperone Network: An Integrative Map of Physical and Genetic Interactions Mediated by the Hsp90 Chaperone. Cell.

