# Supplementary material for: Comparative proteomic analysis revealed complex responses to classical/novel duck reovirus infections in Cairna moschata
Source: Sci Rep. 2018 Jul 4;8:10079. doi: 10.1038/s41598-018-28499-3 (PMC6031628; doi:10.1038/s41598-018-28499-3)
Supplement: Supplementary file 1 — Supplementary Information [file 41598_2018_28499_MOESM1_ESM.pdf]

Comparative proteomic analysis revealed complex responses to classical/novel duck reovirus infections in *Cairna moschata*

Tao Yun, Jionggang Hua, Weicheng Ye, Bin Yu, Liu Chen, Zheng Ni, Cun Zhang *

Institute of Animal Husbandry and Veterinary Sciences, Zhejiang Academy of Agricultural Sciences, Hangzhou 310021, China

* Corresponding author:

Cun Zhang

Email Address: zhangcun@aliyun.com

Table S2 The differential expressed proteins under CDRV infection.

Protein accession	Protein description	CDRV vs Con Ratio	Regulated Type	P value
U3IL38	Malic enzyme	0.212	Down	1.78E-04
R0KBE2	Fatty acid synthase (Fragment)	0.220	Down	1.38E-05
U3IQX8	Acetoacetyl-CoA synthetase (Fragment)	0.238	Down	4.33E-02
A0A0H3U2H5	SCD1	0.247	Down	8.21E-03
U3J276	Biotin--protein ligase (Fragment)	0.303	Down	1.71E-04
Q38HW7	Thyroid hormone responsive Spot 14 beta	0.305	Down	8.12E-03
U3I8S1	Long-chain-fatty-acid--CoA ligase ACSBG2 (Fragment)	0.311	Down	3.24E-03
U3IJJ2	Perilipin	0.316	Down	1.91E-02
R0JKM2	Catechol O-methyltransferase (Fragment)	0.346	Down	2.29E-03
U3IH42	Acetyl-CoA carboxylase	0.354	Down	1.15E-03
U3IST0	Cytochrome P450 2G19	0.364	Down	6.96E-03
U3IHK2	Bifunctional ATP-dependent dihydroxyacetone kinase/FAD-AMP lyase (Cyclizing) (Fragment)	0.366	Down	1.85E-04
U3J925	ATP-citrate synthase	0.368	Down	2.05E-03
R0JJ64	Cytochrome P450 2H1 (Fragment)	0.373	Down	2.13E-03
U3J8P1	Sulfotransferase	0.391	Down	9.96E-03
U3J6U7	"Pyruvate dehydrogenase (Acetyl-transferring) kinase isozyme 1, isoform X1 "	0.395	Down	2.61E-02
U3I031	Glucose-6-phosphate 1-dehydrogenase	0.409	Down	1.36E-03
U3IVG9	Putative hexokinase HKDC1	0.419	Down	2.58E-02
U3J1Q5	Isopentenyl-diphosphate Delta-isomerase 1 (Fragment)	0.427	Down	1.08E-03
U3IU92	Albumin	0.451	Down	4.42E-02
R0K2S6	Epoxide hydrolase 1 (Fragment)	0.463	Down	2.44E-02
R0K523	UPF0131 protein (Fragment)	0.469	Down	3.38E-03
U3IFH4	"6-phosphogluconate dehydrogenase, decarboxylating "	0.471	Down	3.30E-03
U3J544	UDP-glucuronosyltransferase	0.476	Down	2.81E-02
R0JJY5	Alpha-2-HS-glycoprotein (Fragment)	0.481	Down	1.49E-02
R0LFS2	24-dehydrocholesterol reductase (Fragment)	0.489	Down	2.63E-02
R0JP84	UDP-glucuronosyltransferase 1-1 (Fragment)	0.489	Down	2.52E-02
U3J057	Phosphatidylethanolamine-binding protein 1 (Fragment)	0.491	Down	5.24E-03
W8P5B2	Solute carrier family 2 facilitated glucose transporter member 2 (Fragment)	0.501	Down	8.18E-03
U3J2I9	Cytoplasmic dynein 1 light intermediate chain 2 (Fragment)	0.501	Down	6.55E-05

U3IQT7	UTP--glucose-1-phosphate uridylyltransferase (Fragment)	0.507	Down	3.51E-02
R0LTK9	"Glycerol-3-phosphate acyltransferase, mitochondrial (Fragment) "	0.511	Down	4.44E-03
R0JF58	"1,4-alpha-glucan-branching enzyme (Fragment) "	0.511	Down	2.53E-03
R0K7M8	Canalicular multispecific organic anion transporter 1 (Fragment)	0.513	Down	1.31E-03
R0M1D2	"1,2-dihydroxy-3-keto-5-methylthiopentene dioxygenase (Fragment) "	0.517	Down	5.00E-04
R0LET4	"Glycogen [starch] synthase, liver (Fragment) "	0.518	Down	7.41E-03
U3J7S6	Cytochrome P450 2K1 (Fragment)	0.522	Down	6.88E-03
U3I3M1	Bifunctional 3'-phosphoadenosine 5'-phosphosulfate synthetase 2 (Fragment)	0.526	Down	5.19E-03
U3IBU4	Vacuolar protein sorting-associated protein 13C (Fragment)	0.530	Down	4.17E-04
U3IQ11	60S ribosomal protein L22-like 1 (Fragment)	0.531	Down	4.80E-03
U3IIW2	Fatty acid desaturase 2	0.542	Down	2.52E-02
U3II57	Cytochrome P450 51A1 (Fragment)	0.562	Down	2.88E-02
U3IEW2	"Pyruvate dehydrogenase E1 component subunit beta, mitochondrial (Fragment) "	0.568	Down	5.10E-03
U3J4C8	"Fatty acid-binding protein, brain (Fragment) "	0.569	Down	2.78E-02
R0KA47	Pyrroline-5-carboxylate reductase 2 (Fragment)	0.578	Down	5.89E-03
U3IQN0	Alanine aminotransferase 2 (Fragment)	0.578	Down	1.60E-03
U3J3J7	3-keto-steroid reductase (Fragment)	0.579	Down	9.55E-05
U3HZB8	UDP-glucuronosyltransferase	0.582	Down	1.63E-02
U3IWX5	Calcium-regulated heat stable protein 1 (Fragment)	0.584	Down	6.45E-03
U3ISI9	Transaldolase	0.585	Down	2.64E-02
R0JXM5	Malate dehydrogenase (Fragment)	0.589	Down	1.27E-02
U3ILL1	Pyruvate dehydrogenase E1 component subunit alpha	0.592	Down	3.98E-03
R0KYB1	Putative saccharopine dehydrogenase (Fragment)	0.595	Down	7.02E-03
R0JWK0	Dihydropyrimidine dehydrogenase [NADP+] (Fragment)	0.598	Down	2.96E-02
U3INK6	Amidophosphoribosyltransferase	0.599	Down	3.06E-03
R0LFU7	UDP-glucuronosyltransferase 1-1 (Fragment)	0.603	Down	1.47E-02
U3IRA6	"Beta,beta-carotene 15,15'-monooxygenase (Fragment) "	0.606	Down	7.98E-03
U3IX02	Arsenite methyltransferase (Fragment)	0.611	Down	1.69E-02
U3IQT2	Bile salt export pump	0.613	Down	1.32E-03
U3IKU2	Non-specific lipid-transfer protein	0.624	Down	1.29E-02
U3ICQ7	Acetyltransferase component of pyruvate dehydrogenase complex	0.628	Down	4.67E-03
U3J671	Short/branched chain specific acyl-CoA	0.628	Down	7.87E-03

	dehydrogenase			
U3IQB4	Hydroxyacid oxidase 2 (Fragment)	0.632	Down	2.99E-02
U3ICK7	Phosphoserine aminotransferase	0.632	Down	2.00E-03
U3IE65	Glutathione transferase omega-1 (Fragment)	0.634	Down	1.49E-02
U3IKR7	10-formyltetrahydrofolate dehydrogenase	0.635	Down	9.40E-04
R0LHU9	Alpha-1-antiproteinase (Fragment)	0.637	Down	1.66E-03
U3I6G0	"Glycine amidinotransferase, mitochondrial (Fragment) "	0.639	Down	1.27E-02
U3ISH9	Catalase	0.643	Down	1.91E-03
U3J788	Tether containing UBX domain for GLUT4 (Fragment)	0.643	Down	1.80E-02
U3INV9	ATP-binding cassette sub-family D member 3 (Fragment)	0.655	Down	1.18E-02
R0LSK5	Aflatoxin B1 aldehyde reductase member 2 (Fragment)	0.657	Down	1.37E-02
U3IPN9	Multifunctional protein ADE2 (Fragment)	0.658	Down	3.43E-02
R0LZS2	Acetyltransferase component of pyruvate dehydrogenase complex (Fragment)	0.660	Down	2.73E-03
U3IUR5	Tyrosine-protein kinase	1.506	Up	4.96E-02
U3IPM4	Hippocalcin-like protein 1	1.506	Up	3.00E-03
U3ITZ5	Calcium and integrin-binding protein 1 (Fragment)	1.507	Up	3.53E-02
U3ICC7	Coronin	1.507	Up	2.75E-02
U3ITQ7	Protein disulfide-isomerase A6 (Fragment)	1.511	Up	1.00E-02
U3I8K1	Putative calreticulin variant 2 (Fragment)	1.511	Up	1.08E-02
U3ITZ9	LDLR chaperone MESD	1.514	Up	3.04E-02
U3IBG7	Sodium channel protein	1.516	Up	9.34E-03
R0KRP3	Zinc finger CCHC domain-containing protein 11 (Fragment)	1.517	Up	1.31E-02
U3IC97	DnaJ subfamily B member 11 (Fragment)	1.520	Up	3.82E-04
R0K012	Activator of 90 kDa heat shock protein ATPase-like protein 2 (Fragment)	1.521	Up	3.25E-02
U3J7P5	C-terminal-binding protein 1 (Fragment)	1.523	Up	5.15E-04
U3IYU5	Cathepsin S (Fragment)	1.525	Up	1.72E-02
R0LEJ9	Golgi SNAP receptor complex member 1 (Fragment)	1.526	Up	1.72E-02
U3I503	Calcium-binding mitochondrial carrier protein Aralar1 (Fragment)	1.529	Up	4.30E-02
R0LIP6	"Beta-1,3-glucosyltransferase (Fragment) "	1.531	Up	2.36E-02
U3IFM7	Periostin (Fragment)	1.531	Up	4.25E-02
U3IK65	B-cell receptor-associated protein 29 (Fragment)	1.531	Up	1.06E-02
U3IGL9	Protein flightless-1 (Fragment)	1.534	Up	1.76E-03
U3I4W0	45 kDa calcium-binding protein	1.535	Up	2.56E-02
U3IJ05	GDP/GTP exchange factor VAV2	1.535	Up	8.11E-04

R0JBS1	"Beta-galactoside alpha-2,6-sialyltransferase 1 (Fragment) "	1.541	Up	1.47E-02
U3I4Y9	YLP motif-containing protein 1 (Fragment)	1.546	Up	4.26E-02
U3I0C5	Plastin-2	1.558	Up	6.85E-03
U3I640	78 kDa glucose-regulated protein	1.559	Up	2.02E-03
R0LWF3	Switch-associated protein 70 (Fragment)	1.561	Up	2.19E-02
U3IFX9	Kininogen-1 (Fragment)	1.562	Up	3.35E-02
U3IDX4	Cytochrome P450 3A4 (Fragment)	1.570	Up	3.75E-03
U3I793	Formin-like protein 1	1.572	Up	1.35E-02
U3IP43	Prolyl 4-hydroxylase subunit alpha-1 (Fragment)	1.576	Up	2.51E-03
U3IAA8	Leucine-rich repeat flightless-interacting protein 2 (Fragment)	1.579	Up	3.08E-03
U3IE04	Segment polarity protein dishevelled homolog DVL-3	1.583	Up	1.28E-02
U3IUW5	DnaJ-like protein subfamily C member 3 (Fragment)	1.583	Up	7.55E-03
U3I6U1	Rho GDP-dissociation inhibitor 2 (Fragment)	1.585	Up	1.47E-02
U3IG19	Endoplasmic reticulum aminopeptidase 1 (Fragment)	1.585	Up	7.05E-05
U3J9T4	Deoxyribonuclease-2-beta (Fragment)	1.585	Up	2.72E-02
U3IHS7	SH3 domain-containing kinase-binding protein 1 (Fragment)	1.591	Up	1.06E-02
U3J1A4	Nuclear factor NF-kappa-B p100 subunit	1.592	Up	1.12E-02
U3IAM8	Transmembrane 9 superfamily member	1.595	Up	2.70E-03
R0M6C3	Tripartite motif-containing protein 47	1.598	Up	1.25E-02
R0JD94	3-ketodihydrosphingosine reductase (Fragment)	1.607	Up	6.57E-03
U3I7D4	Ena/VASP-like protein	1.610	Up	3.00E-02
R0K642	Ladinin-1	1.612	Up	3.88E-03
U3IFK1	Actin-related protein 2/3 complex subunit 1B (Fragment)	1.612	Up	3.41E-02
U3IMQ6	Leukocyte common antigen (Fragment)	1.620	Up	2.86E-02
R0K171	Integrin beta (Fragment)	1.624	Up	3.94E-02
U3HZL5	Peptidyl-prolyl cis-trans isomerase	1.627	Up	7.08E-03
U3I523	Glucocorticoid receptor DNA-binding factor 1 (Fragment)	1.628	Up	3.57E-04
U3IWS8	Hexokinase2	1.638	Up	4.04E-02
U3HZG6	Chloride intracellular channel protein	1.642	Up	1.53E-02
R0KBX6	Protein disulfide-isomerase (Fragment)	1.645	Up	2.66E-03
U3IC71	Pyridoxal-dependent decarboxylase domain-containing protein 1 (Fragment)	1.645	Up	4.58E-03
U3ID52	Sorcin (Fragment)	1.647	Up	3.17E-02
U3IIY3	Complement C5 (Fragment)	1.647	Up	1.26E-04
R4HH66	Desmin (Fragment)	1.654	Up	3.22E-02

U3IY10	Tropomyosin alpha-3 chain (Fragment)	1.654	Up	2.22E-04
R0LCK1	Dipeptidyl-peptidase 1 (Fragment)	1.655	Up	8.61E-04
U3IQR4	GDH/6PGL endoplasmic bifunctional protein (Fragment)	1.660	Up	2.74E-02
U3IB49	Leukocyte cell-derived chemotaxin 2	1.674	Up	1.19E-02
U3I3A3	Polypeptide N-acetylgalactosaminyltransferase	1.676	Up	2.92E-04
U3J9W7	N-acylethanolamine-hydrolyzing acid amidase (Fragment)	1.680	Up	1.05E-02
U3HZJ9	Ribosome-binding protein 1	1.689	Up	2.69E-03
U3I728	Tudor domain-containing protein 7	1.690	Up	7.63E-04
R0JUP5	"Arylamine N-acetyltransferase, pineal gland isozyme NAT-3 (Fragment) "	1.695	Up	1.87E-02
U3I693	Acetyl-coenzyme A synthetase	1.705	Up	4.31E-03
R0L6A3	Bifunctional 3'-phosphoadenosine 5'-phosphosulfate synthetase 1 (Fragment)	1.705	Up	1.22E-02
U3I8R2	Complement C4 (Fragment)	1.719	Up	1.58E-02
U3IL04	Torsin-1B	1.723	Up	1.99E-02
U3IWC6	E3 ISG15--protein ligase HERC5 (Fragment)	1.733	Up	9.48E-03
U3I2Q9	Transporter associated with antigen processing 1	1.736	Up	8.95E-03
U3J9B8	Collagen alpha-6(VI) chain	1.739	Up	1.07E-02
U3IVN5	CCAAT/enhancer-binding protein beta (Fragment)	1.740	Up	4.99E-03
U3ISI5	Leucine-rich repeat flightless-interacting protein 2 (Fragment)	1.745	Up	2.85E-02
R0JS80	Fibrinogen beta chain (Fragment)	1.748	Up	1.66E-02
P00706	Lysozyme C-3	1.752	Up	4.03E-03
A0A0C4K5J8	Interferon-induced transmembrane protein 2	1.753	Up	2.75E-02
R0JWF8	Ribonuclease T2 (Fragment)	1.754	Up	4.04E-03
U3IXR6	Phospholipase D1	1.755	Up	1.04E-02
R0L541	Peroxisomal proliferator-activated receptor A-interacting complex 285 kDa protein (Fragment)	1.759	Up	2.52E-03
U3IMY2	EH domain-containing protein 4 (Fragment)	1.760	Up	1.50E-02
R0KUV7	Calreticulin (Fragment)	1.762	Up	1.83E-02
U3IKF3	Complement component C8 beta chain (Fragment)	1.771	Up	1.10E-02
U3HZV7	Ovoinhibitor	1.783	Up	3.44E-02
R0LE49	"Fatty acid-binding protein, liver (Fragment) "	1.800	Up	1.43E-02
U3I9S0	Tropomyosin beta chain isoform 2	1.802	Up	3.29E-03
U3IZH0	Transgelin-2 (Fragment)	1.807	Up	1.09E-02
U3IUA7	Complement factor H (Fragment)	1.812	Up	6.05E-04
R0L013	Protein FADD (Fragment)	1.816	Up	2.13E-02
U3IN55	Complement component C9 (Fragment)	1.823	Up	1.43E-03
U3INH5	Complement component C7 (Fragment)	1.826	Up	6.50E-05
U3IVR6	Syntaxin-17	1.830	Up	2.18E-02
U3J4X1	Endoplasmin	1.832	Up	1.59E-03

U3J0M7	Signal transducer and activator of transcription 1 (Fragment)	1.832	Up	5.62E-04
U3IUL2	Phosphoinositide 3-kinase adapter protein 1 (Fragment)	1.834	Up	6.31E-04
R0K5F7	OTU domain-containing protein 4 (Fragment)	1.854	Up	1.37E-02
U3IPW6	Interferon-induced guanylate-binding protein 1 (Fragment)	1.861	Up	3.61E-02
U3IE41	PX domain-containing protein kinase-like (Fragment)	1.866	Up	6.69E-04
U3IMH2	Pantetheinase (Fragment)	1.882	Up	1.44E-02
R0M8K4	Toll-like receptor 3 (Fragment)	1.884	Up	1.98E-03
U3I577	RING finger protein 213 (Fragment)	1.889	Up	6.03E-03
U3IAG4	Optineurin (Fragment)	1.892	Up	1.31E-02
U3IYM9	26S proteasome non-ATPase regulatory subunit 3	1.897	Up	2.05E-02
R0KZL1	Calreticulin (Fragment)	1.899	Up	7.88E-03
U3J989	"Beta-1,4-galactosyltransferase 1 (Fragment) "	1.909	Up	1.15E-03
U3J3R4	Cap-specific mRNA (Nucleoside-2'-O-)-methyltransferase 1	1.917	Up	1.42E-02
U3J4N1	Deoxynucleoside triphosphate triphosphohydrolase SAMHD1	1.921	Up	4.14E-03
U3ID07	Complement C1s subcomponent	1.959	Up	1.43E-03
R0LEU9	"Chromosome 17 SCAF14597, whole genome shotgun sequence "	1.964	Up	3.27E-02
U3IGH7	Alpha-2-macroglobulin (Fragment)	1.977	Up	8.82E-03
U3J0V8	Signal transducer and activator of transcription 1	1.993	Up	5.16E-04
R0LYX6	Complement component receptor 1-like protein (Fragment)	2.005	Up	3.80E-03
R0K9F9	GMP synthase [glutamine-hydrolyzing] (Fragment)	2.006	Up	1.12E-02
U3IRC3	Poly [ADP-ribose] polymerase	2.013	Up	1.09E-03
U3IKH3	Complement component C8 alpha chain (Fragment)	2.018	Up	1.29E-02
U3IGG3	Acetyl-coenzyme A transporter 1 (Fragment)	2.019	Up	8.50E-03
U3ICR2	Collectin-46 (Fragment)	2.021	Up	2.61E-03
U3J6P0	Complement C3	2.054	Up	7.71E-04
U3ID39	Carbonic anhydrase-related protein (Fragment)	2.055	Up	1.72E-02
P00705	Lysozyme C-1	2.088	Up	1.10E-03
R0JF61	CD5 antigen-like (Fragment)	2.096	Up	1.63E-02
U3IN20	Hemopexin	2.121	Up	8.01E-03
U3I028	Sterile alpha motif domain-containing protein 9-like (Fragment)	2.124	Up	1.17E-03
R0LGA4	Poly [ADP-ribose] polymerase (Fragment)	2.125	Up	5.05E-04
R0JRR9	Ras-related protein Rab-32 (Fragment)	2.141	Up	1.23E-03
U3I7F0	MHC-linked complement C4	2.143	Up	3.32E-02

U3I6G9	Opioid growth factor receptor (Fragment)	2.166	Up	5.52E-04
U3IY90	Serpin B10 (Fragment)	2.168	Up	1.54E-02
U3IHU6	GMP synthase [glutamine-hydrolyzing] (Fragment)	2.179	Up	9.60E-03
U3IIT1	Ubl carboxyl-terminal hydrolase 18 (Fragment)	2.217	Up	3.62E-02
R0JI19	ATP-binding cassette sub-family A member 8 (Fragment)	2.221	Up	3.24E-02
R0KF58	Transmembrane protein 85	2.224	Up	1.45E-02
U3I742	Alpha-2-macroglobulin (Fragment)	2.263	Up	1.05E-02
S4SM19	ATP-dependent RNA helicase (Fragment)	2.286	Up	1.87E-03
U3IEV3	Tyrosine-protein kinase	2.292	Up	2.64E-03
R0LGG0	Complement C1q subcomponent subunit A (Fragment)	2.293	Up	2.70E-04
U3ITB1	Alpha-2-macroglobulin-like 1 (Fragment)	2.351	Up	1.98E-02
V9HXS8	MHC class I antigen heavy chain (Fragment)	2.383	Up	4.92E-02
A0A0M3RQ94	Retinoic acid inducible protein I	2.392	Up	4.35E-02
U3I8M2	Interferon-induced 35 kDa protein	2.394	Up	8.69E-04
R0JNZ4	Macrophage-capping protein (Fragment)	2.411	Up	9.21E-03
Q2VQY9	MHC class I antigen alpha chain	2.448	Up	7.87E-03
R0LW73	Complement C1q subcomponent subunit B (Fragment)	2.458	Up	2.12E-04
A0A0M4S8A2	Double-stranded RNA-dependent protein kinase	2.473	Up	6.52E-05
U3I8Y0	Normal mucosa of esophagus-specific 1 (Fragment)	2.497	Up	4.31E-02
U3IGY9	"Solute carrier family 2, facilitated glucose transporter member 6 (Fragment) "	2.525	Up	2.37E-03
R0JBX5	Homeodomain-only protein (Fragment)	2.530	Up	9.83E-03
U3IGN0	N-myc-interactor (Fragment)	2.574	Up	2.15E-03
U3I1F8	Hemopexin (Fragment)	2.662	Up	2.58E-03
U3HZR3	Interferon-induced very large GTPase 1 (Fragment)	2.754	Up	3.93E-03
Q14U75	Beta-2-microglobulin	2.882	Up	3.30E-03
U3I321	"2',3'-cyclic-nucleotide 3'-phosphodiesterase (Fragment) "	2.885	Up	8.52E-03
R0LDN0	Deleted in malignant brain tumors 1 protein	2.904	Up	7.68E-04
R0KUN6	Protein-glutamine gamma-glutamyltransferase E (Fragment)	2.965	Up	1.16E-02
R0JXF2	Putative ATP-dependent RNA helicase DDX60 (Fragment)	2.993	Up	4.79E-03
U3IEV1	Nicotinamide phosphoribosyltransferase (Fragment)	3.005	Up	1.92E-03
U3J8T9	L-amino-acid oxidase (Fragment)	3.048	Up	2.87E-02
R0JS12	Serine protease inhibitor A3M (Fragment)	3.114	Up	1.43E-02
P68494	Metallothionein	3.124	Up	1.73E-02
R0LJZ9	"UMP-CMP kinase 2, mitochondrial (Fragment) "	3.188	Up	1.56E-03

U3IRN3	Protein MRP-126	3.207	Up	3.46E-02
U3ITA4	Argininosuccinate synthase	3.407	Up	7.36E-04
R0K9R1	BCL2-like protein 15 (Fragment)	3.516	Up	5.94E-03
U3IJS3	Ceruloplasmin (Fragment)	3.676	Up	7.44E-03
P33238	Interferon-induced GTP-binding protein Mx	4.535	Up	2.47E-02
R0M3E0	Pantetheinase (Fragment)	4.774	Up	1.47E-02
P02740	Serum amyloid A protein	4.850	Up	1.53E-02
U3IC83	Serum amyloid A protein	7.546	Up	2.87E-03
U3I466	Alpha-1-acid glycoprotein	8.498	Up	5.38E-03

Table S3 The differential expressed proteins under NDRV infection.

Protein accession	Protein description	NDRVvsCon Ratio	Regulated Type	P value
U3IL38	Malic enzyme	0.199	Down	1.92E-03
R0KBE2	Fatty acid synthase (Fragment)	0.218	Down	8.50E-05
A0A0H3U2H5	SCD1	0.228	Down	1.40E-02
U3IQX8	Acetoacetyl-CoA synthetase (Fragment)	0.270	Down	3.50E-02
R0JJ64	Cytochrome P450 2H1 (Fragment)	0.281	Down	1.28E-04
U3J276	Biotin--protein ligase (Fragment)	0.298	Down	1.48E-03
Q38HW7	Thyroid hormone responsive Spot 14 beta	0.301	Down	3.91E-03
U3IH42	Acetyl-CoA carboxylase	0.331	Down	5.36E-03
U3J8P1	Sulfotransferase	0.344	Down	1.15E-02
U3I8S1	Long-chain-fatty-acid--CoA ligase ACSBG2 (Fragment)	0.346	Down	4.43E-03
R0JKM2	Catechol O-methyltransferase (Fragment)	0.355	Down	1.85E-03
R0K2S6	Epoxide hydrolase 1 (Fragment)	0.356	Down	1.55E-02
U3J925	ATP-citrate synthase	0.356	Down	4.77E-03
U3IJJ2	Perilipin	0.366	Down	1.14E-02
U3J6U7	"Pyruvate dehydrogenase (Acetyl-transferring) kinase isozyme 1, isoform X1 "	0.398	Down	2.09E-02
U3IVG9	Putative hexokinase HKDC1	0.420	Down	3.69E-02
R0JJY5	Alpha-2-HS-glycoprotein (Fragment)	0.439	Down	2.38E-02
U3I3M1	Bifunctional 3'-phosphoadenosine 5'-phosphosulfate synthetase 2 (Fragment)	0.440	Down	9.15E-03
R0M1D2	"1,2-dihydroxy-3-keto-5-methylthiopentene dioxygenase (Fragment) "	0.442	Down	6.79E-04
U3I4K0	Protein NipSnap-like protein 3A (Fragment)	0.442	Down	4.85E-02
U3I031	Glucose-6-phosphate 1-dehydrogenase	0.449	Down	5.16E-03
U3IHK2	Bifunctional ATP-dependent dihydroxyacetone kinase/FAD-AMP lyase (Cyclizing) (Fragment)	0.452	Down	2.28E-03
U3IST0	Cytochrome P450 2G19	0.457	Down	1.11E-02
U3IUH7	Alpha-tocopherol transfer protein (Fragment)	0.464	Down	5.11E-03
W8P5B2	Solute carrier family 2 facilitated glucose transporter member 2 (Fragment)	0.471	Down	1.83E-02
U3J057	Phosphatidylethanolamine-binding protein 1 (Fragment)	0.472	Down	1.56E-03
U3IFH4	"6-phosphogluconate dehydrogenase, decarboxylating "	0.477	Down	2.78E-03
R0LTK9	"Glycerol-3-phosphate acyltransferase, mitochondrial (Fragment) "	0.478	Down	9.58E-03
U3J4C8	"Fatty acid-binding protein, brain (Fragment) "	0.480	Down	9.74E-03

U3J2I9	Cytoplasmic dynein 1 light intermediate chain 2 (Fragment)	0.481	Down	6.27E-05
U3ICK7	Phosphoserine aminotransferase	0.483	Down	1.36E-04
U3J1Q5	Isopentenyl-diphosphate Delta-isomerase 1 (Fragment)	0.485	Down	4.56E-03
R0K523	UPF0131 protein (Fragment)	0.489	Down	4.00E-03
U3IQT7	UTP--glucose-1-phosphate uridylyltransferase (Fragment)	0.493	Down	3.85E-02
U3J7S6	Cytochrome P450 2K1 (Fragment)	0.517	Down	4.80E-03
U3IBU4	Vacuolar protein sorting-associated protein 13C (Fragment)	0.519	Down	1.34E-03
U3J671	Short/branched chain specific acyl-CoA dehydrogenase	0.522	Down	1.39E-02
R0JP84	UDP-glucuronosyltransferase 1-1 (Fragment)	0.528	Down	3.39E-02
R0JNR2	Solute carrier organic anion transporter family member (Fragment)	0.531	Down	4.55E-02
U3IDQ1	Acyl-coenzyme A oxidase	0.532	Down	3.43E-02
U3IX02	Arsenite methyltransferase (Fragment)	0.537	Down	2.61E-03
U3IIS9	Phosphoribosylformylglycinamidine synthase	0.541	Down	1.02E-02
U3J383	Phosphoglucomutase-1 (Fragment)	0.546	Down	7.13E-03
U3I700	Dimethylaniline monooxygenase [N-oxide-forming]	0.548	Down	4.81E-02
U3IEW2	"Pyruvate dehydrogenase E1 component subunit beta, mitochondrial (Fragment) "	0.551	Down	1.26E-02
R0JXM5	Malate dehydrogenase (Fragment)	0.556	Down	1.34E-02
R0JF58	"1,4-alpha-glucan-branching enzyme (Fragment) "	0.557	Down	1.31E-02
U3ILL1	Pyruvate dehydrogenase E1 component subunit alpha	0.558	Down	8.86E-03
U3I535	3-oxo-5-beta-steroid 4-dehydrogenase-like isoform X1	0.562	Down	2.69E-03
R0LBD9	Sulfotransferase (Fragment)	0.562	Down	8.12E-03
R0JWK0	Dihydropyrimidine dehydrogenase [NADP+] (Fragment)	0.564	Down	1.29E-02
R0JLU6	DEP domain-containing protein 6 (Fragment)	0.567	Down	3.27E-02
R0KA47	Pyrroline-5-carboxylate reductase 2 (Fragment)	0.569	Down	3.35E-02
U3IQT2	Bile salt export pump	0.570	Down	2.34E-03
R0L0L6	UDP-glucuronosyltransferase 1-1 (Fragment)	0.570	Down	2.22E-02
U3IHC5	Chloride intracellular channel protein	0.570	Down	2.41E-02
U3IUA0	Phenylalanine hydroxylase	0.571	Down	1.03E-02
U3IWX5	Calcium-regulated heat stable protein 1 (Fragment)	0.573	Down	1.75E-02
U3J2H8	"Fructose-1,6-bisphosphatase 1 (Fragment) "	0.577	Down	1.53E-03

U3IQ11	60S ribosomal protein L22-like 1 (Fragment)	0.580	Down	2.50E-03
U3ISH9	Catalase	0.580	Down	1.18E-03
U3IRA6	"Beta,beta-carotene 15,15'-monooxygenase (Fragment) "	0.582	Down	1.55E-02
R0KYB1	Putative saccharopine dehydrogenase (Fragment)	0.588	Down	1.56E-02
R0LHU9	Alpha-1-antiproteinase (Fragment)	0.589	Down	5.46E-03
R0LET4	"Glycogen [starch] synthase, liver (Fragment) "	0.596	Down	8.22E-03
U3ICQ7	Acetyltransferase component of pyruvate dehydrogenase complex	0.601	Down	1.11E-03
R0K7M8	Canalicular multispecific organic anion transporter 1 (Fragment)	0.602	Down	2.48E-03
U3INV9	ATP-binding cassette sub-family D member 3 (Fragment)	0.603	Down	2.74E-03
R0LSD9	Phosphotriesterase-related protein (Fragment)	0.605	Down	4.29E-03
U3IGJ7	Beta-lactamase-like protein 2 (Fragment)	0.605	Down	5.76E-04
U3ISI9	Transaldolase	0.606	Down	2.20E-02
U3J1L9	Glycogen debranching enzyme (Fragment)	0.606	Down	2.08E-02
U3INK6	Amidophosphoribosyltransferase	0.610	Down	3.45E-03
U3IHG8	Fructose-bisphosphate aldolase	0.614	Down	1.13E-02
U3I6G0	"Glycine amidinotransferase, mitochondrial (Fragment) "	0.618	Down	1.01E-02
U3HZB8	UDP-glucuronosyltransferase	0.619	Down	1.76E-02
U3IX07	Stabilin-2 (Fragment)	0.621	Down	3.52E-02
U3IWL5	Protein Z-dependent protease inhibitor (Fragment)	0.624	Down	4.36E-02
U3J4M3	"Acyl-coenzyme A synthetase ACSM4, mitochondrial (Fragment) "	0.627	Down	2.35E-03
D7REA1	Basic fatty acid binding protein	0.633	Down	1.83E-02
U3IUY3	Serine hydroxymethyltransferase	0.635	Down	4.63E-02
U3IPN9	Multifunctional protein ADE2 (Fragment)	0.638	Down	2.11E-02
U3IAM4	Cytosolic purine 5'-nucleotidase	0.641	Down	8.72E-03
U3IJE1	Dihydropteridine reductase	0.643	Down	1.85E-03
U3IGS6	Stabilin-1 (Fragment)	0.644	Down	7.02E-05
R0LSK5	Aflatoxin B1 aldehyde reductase member 2 (Fragment)	0.645	Down	2.63E-02
R0K265	Epoxide hydrolase 1 (Fragment)	0.648	Down	1.11E-03
U3IE65	Glutathione transferase omega-1 (Fragment)	0.650	Down	2.70E-02
R0LZS2	Acetyltransferase component of pyruvate dehydrogenase complex (Fragment)	0.653	Down	6.26E-03
R0K747	Macrophage mannose receptor 1 (Fragment)	0.653	Down	1.15E-02
U3IFA7	NADH-cytochrome b5 reductase	0.654	Down	2.76E-02
U3IV51	Solute carrier family 40 member 1 (Fragment)	0.656	Down	4.42E-02
R0L6Q1	Delta-1-pyrroline-5-carboxylate synthetase	0.657	Down	1.49E-02

	(Fragment)			
U3I814	Putative imidazolonepropionase (Fragment)	0.658	Down	7.79E-04
U3J796	Retinol-binding protein 4 (Fragment)	0.660	Down	1.10E-02
R0LTM3	Long-chain-fatty-acid--CoA ligase 5 (Fragment)	0.660	Down	1.91E-02
R0L4D8	Cytoplasmic dynein 1 heavy chain 1 (Fragment)	0.663	Down	2.15E-03
U3J788	Tether containing UBX domain for GLUT4 (Fragment)	0.666	Down	2.90E-02
R0JP74	UDP-glucuronosyltransferase 1-1 (Fragment)	0.667	Down	3.73E-02
R0KDK0	Fructose-bisphosphate aldolase (Fragment)	1.500	Up	2.29E-02
U3IBI9	Nuclear mitotic apparatus protein	1.502	Up	1.33E-02
R0LEJ9	Golgi SNAP receptor complex member 1 (Fragment)	1.505	Up	1.38E-02
U3IFS3	Dedicator of cytokinesis protein 10 (Fragment)	1.506	Up	4.27E-02
R0LC19	Heat shock 70 kDa protein (Fragment)	1.510	Up	2.75E-02
U3I8W8	RGD-CAP	1.510	Up	1.72E-03
A0A023ND52	DEAD box polypeptide 41 (Fragment)	1.510	Up	2.79E-02
U3J851	Ovotransferrin (Fragment)	1.512	Up	4.03E-02
U3I674	Armadillo repeat-containing protein 8-like isoform X1	1.514	Up	1.19E-02
R0LWF3	Switch-associated protein 70 (Fragment)	1.515	Up	3.75E-02
U3HZL5	Peptidyl-prolyl cis-trans isomerase	1.517	Up	1.89E-02
R0JJW5	Collagen alpha-2(VI) chain (Fragment)	1.519	Up	2.29E-02
U3I523	Glucocorticoid receptor DNA-binding factor 1 (Fragment)	1.521	Up	1.99E-03
U3J0L3	Syntaxin-2 (Fragment)	1.522	Up	3.66E-02
R0LQT9	Lymphoid-specific helicase (Fragment)	1.522	Up	2.27E-02
U3IAM8	Transmembrane 9 superfamily member	1.522	Up	3.28E-03
U3I5F7	Myosin-If (Fragment)	1.522	Up	4.24E-03
U3I4Q7	Putative phospholipase B-like 1 (Fragment)	1.524	Up	1.61E-02
U3IBL6	Neutrophil cytosol factor 1 (Fragment)	1.524	Up	2.91E-02
U3IHY0	Protein FAM65B (Fragment)	1.525	Up	4.73E-02
U3ITC2	"ES1 protein-like protein, mitochondrial (Fragment) "	1.525	Up	4.70E-02
R0LAM5	[Protein ADP-ribosylarginine] hydrolase (Fragment)	1.528	Up	6.79E-03
R0JY18	Chromosome 17 open reading frame 62	1.528	Up	3.14E-03
U3IE04	Segment polarity protein dishevelled homolog DVL-3	1.529	Up	1.06E-02
U3IQR4	GDH/6PGL endoplasmic bifunctional protein (Fragment)	1.530	Up	1.75E-02
R0KY51	Cartilage oligomeric matrix protein (Fragment)	1.531	Up	3.20E-02
U3I640	78 kDa glucose-regulated protein	1.533	Up	5.65E-03
R0M528	Protein phosphatase 1 regulatory subunit 12A	1.538	Up	4.95E-02

	(Fragment)			
R0KG68	Nuclear fragile X mental retardation-interacting protein 1 (Fragment)	1.539	Up	8.89E-03
R0KRP3	Zinc finger CCHC domain-containing protein 11 (Fragment)	1.541	Up	7.84E-03
U3IY10	Tropomyosin alpha-3 chain (Fragment)	1.543	Up	5.98E-03
R0JBS1	"Beta-galactoside alpha-2,6-sialyltransferase 1 (Fragment) "	1.545	Up	3.45E-02
U3HZJ9	Ribosome-binding protein 1	1.547	Up	1.40E-03
R0K1Z7	Plasminogen (Fragment)	1.549	Up	6.00E-04
U3IUW5	DnaJ-like protein subfamily C member 3 (Fragment)	1.550	Up	3.98E-03
U3ICM6	Gelsolin (Fragment)	1.552	Up	3.15E-03
U3IES4	Complement C1r subcomponent (Fragment)	1.554	Up	2.10E-02
R0JUP5	"Arylamine N-acetyltransferase, pineal gland isozyme NAT-3 (Fragment) "	1.555	Up	1.84E-02
U3J8F2	Cysteine-rich with EGF-like domain protein 2-A (Fragment)	1.556	Up	3.92E-02
U3J380	Leucyl-cystinyl aminopeptidase (Fragment)	1.557	Up	5.85E-03
U3IX09	Rho-related GTP-binding protein RhoG	1.560	Up	1.01E-02
U3IUL2	Phosphoinositide 3-kinase adapter protein 1 (Fragment)	1.565	Up	1.38E-02
U3IYH7	KDEL motif-containing protein 2 (Fragment)	1.565	Up	1.87E-02
R0K642	Ladinin-1	1.566	Up	3.84E-03
U3J0L6	PRA1 family protein 3 (Fragment)	1.570	Up	1.41E-02
R0L3X3	UPF0235 protein C15orf40-like protein (Fragment)	1.571	Up	1.29E-02
U3I3X8	Annexin	1.571	Up	5.90E-03
U3I2D5	BAG family molecular chaperone regulator 5	1.575	Up	2.36E-02
R0K2W7	"Glycerol-3-phosphate dehydrogenase, mitochondrial (Fragment) "	1.580	Up	1.75E-02
U3IHG0	Prostaglandin F2 receptor negative regulator (Fragment)	1.584	Up	3.07E-03
R0K012	Activator of 90 kDa heat shock protein ATPase-like protein 2 (Fragment)	1.585	Up	9.07E-03
U3IVN5	CCAAT/enhancer-binding protein beta (Fragment)	1.587	Up	3.72E-03
R0L636	High mobility group protein B2 (Fragment)	1.588	Up	2.08E-02
U3IQX0	Chromatin target of PRMT1 protein (Fragment)	1.595	Up	2.15E-02
R0K9F9	GMP synthase [glutamine-hydrolyzing] (Fragment)	1.596	Up	2.24E-02
U3I9S0	Tropomyosin beta chain isoform 2	1.598	Up	9.56E-04
U3I6N5	PRKR-interacting protein 1-like protein	1.606	Up	4.12E-03

	(Fragment)			
U3HZN4	"Ras association (RalGDS/AF-6) and pleckstrin homology domains 1, isoform CRA_b "	1.610	Up	3.99E-03
U3IUA7	Complement factor H (Fragment)	1.613	Up	7.77E-03
U3IHS7	SH3 domain-containing kinase-binding protein 1 (Fragment)	1.616	Up	1.02E-02
U3ITZ5	Calcium and integrin-binding protein 1 (Fragment)	1.616	Up	1.07E-02
U3IUP4	NFX1-type zinc finger-containing protein 1 (Fragment)	1.616	Up	3.82E-03
U3IFK1	Actin-related protein 2/3 complex subunit 1B (Fragment)	1.617	Up	8.49E-03
U3I7S4	Zyxin (Fragment)	1.619	Up	6.75E-04
U3I4Y9	YLP motif-containing protein 1 (Fragment)	1.624	Up	7.19E-03
U3J1A4	Nuclear factor NF-kappa-B p100 subunit	1.625	Up	1.26E-02
U3I410	Protein tweety homolog	1.626	Up	1.28E-02
R0KWN0	Phosphatidylinositol-5-phosphate 4-kinase type-2 alpha (Fragment)	1.627	Up	9.12E-03
U3IN50	Cytochrome b-245 heavy chain (Fragment)	1.637	Up	2.61E-02
U3IHU6	GMP synthase [glutamine-hydrolyzing] (Fragment)	1.639	Up	1.36E-02
U3J9B8	Collagen alpha-6(VI) chain	1.641	Up	1.68E-03
R0KBX6	Protein disulfide-isomerase (Fragment)	1.646	Up	2.50E-03
U3J9W7	N-acylethanolamine-hydrolyzing acid amidase (Fragment)	1.649	Up	2.98E-02
U3J4N1	Deoxynucleoside triphosphate triphosphohydrolase SAMHD1	1.650	Up	1.13E-02
U3IUI2	Septin-6 (Fragment)	1.653	Up	2.66E-02
U3IGG3	Acetyl-coenzyme A transporter 1 (Fragment)	1.661	Up	7.96E-03
U3I5A6	Coagulation factor X	1.665	Up	1.12E-02
U3J9T4	Deoxyribonuclease-2-beta (Fragment)	1.666	Up	3.71E-02
U3IF30	STE20-like serine/threonine-protein kinase (Fragment)	1.667	Up	2.92E-03
U3J9E5	CPEB-associated factor Maskin (Fragment)	1.675	Up	1.41E-02
U3J895	Ras association domain-containing protein 2	1.679	Up	1.35E-02
U3IFX9	Kininogen-1 (Fragment)	1.682	Up	1.53E-02
U3HZG6	Chloride intracellular channel protein	1.698	Up	3.37E-03
R4HH66	Desmin (Fragment)	1.698	Up	1.82E-02
U3IC71	Pyridoxal-dependent decarboxylase domain-containing protein 1 (Fragment)	1.699	Up	1.34E-03
U3INN0	Pleckstrin (Fragment)	1.700	Up	1.40E-02
U3IL04	Torsin-1B	1.700	Up	2.27E-03
U3IN20	Hemopexin	1.702	Up	2.93E-02

U3INM1	Cortactin	1.702	Up	5.21E-03
U3IGH7	Alpha-2-macroglobulin (Fragment)	1.704	Up	2.21E-02
U3J5Y2	Breast cancer anti-estrogen resistance protein 1 (Fragment)	1.704	Up	2.69E-02
U3ICC7	Coronin	1.704	Up	1.77E-02
U3ISI5	Leucine-rich repeat flightless-interacting protein 2 (Fragment)	1.710	Up	2.63E-02
R0JI60	Lysophosphatidylcholine acyltransferase 2 (Fragment)	1.714	Up	4.00E-02
U3I7D6	Cytoglobin (Fragment)	1.732	Up	3.40E-03
U3IRD0	"Lamina-associated polypeptide 2, isoforms beta/gamma (Fragment) "	1.738	Up	3.67E-03
U3I577	RING finger protein 213 (Fragment)	1.744	Up	2.34E-02
R0JLG7	Rho guanine nucleotide exchange factor 6 (Fragment)	1.749	Up	7.60E-04
U3IIY3	Complement C5 (Fragment)	1.749	Up	1.71E-03
R0LK18	Tudor domain-containing protein 3 (Fragment)	1.759	Up	3.33E-02
U3J4X1	Endoplasmin	1.760	Up	6.40E-03
U3IFA9	Serine/threonine-protein kinase VRK1 (Fragment)	1.764	Up	1.37E-02
U3IE41	PX domain-containing protein kinase-like (Fragment)	1.765	Up	6.24E-05
U3IIY7	Cytochrome P450 3A24 (Fragment)	1.769	Up	9.05E-03
U3IV25	Zinc finger Ran-binding domain-containing protein 2	1.769	Up	2.58E-02
U3ICR2	Collectin-46 (Fragment)	1.777	Up	1.48E-02
R0M6C3	Tripartite motif-containing protein 47	1.786	Up	4.94E-03
U3IMQ6	Leukocyte common antigen (Fragment)	1.787	Up	1.43E-02
U3IP43	Prolyl 4-hydroxylase subunit alpha-1 (Fragment)	1.787	Up	5.38E-03
U3ILA8	Torsin-2A (Fragment)	1.788	Up	1.69E-03
V9HXQ3	MHC class I antigen heavy chain (Fragment)	1.791	Up	2.85E-02
R0L6A3	Bifunctional 3'-phosphoadenosine 5'-phosphosulfate synthetase 1 (Fragment)	1.798	Up	8.62E-03
A0A0C4K5J8	Interferon-induced transmembrane protein 2	1.800	Up	1.72E-02
U3I0F9	Pyruvate kinase	1.802	Up	6.97E-03
R0KUF6	Uncharacterized protein	1.805	Up	3.75E-03
U3I693	Acetyl-coenzyme A synthetase	1.814	Up	3.51E-03
R0M8K4	Toll-like receptor 3 (Fragment)	1.823	Up	2.28E-03
U3IWQ9	Vitamin K-dependent protein S (Fragment)	1.824	Up	3.07E-03
U3I596	ATP-binding cassette sub-family A member 3 (Fragment)	1.826	Up	1.63E-02
U3J6P0	Complement C3	1.848	Up	1.25E-03
U3IRP5	Plasminogen	1.857	Up	8.28E-03

R0L013	Protein FADD (Fragment)	1.857	Up	2.90E-03
U3I728	Tudor domain-containing protein 7	1.862	Up	1.27E-03
U3J370	SUN domain-containing protein 2 (Fragment)	1.871	Up	1.34E-03
R0K171	Integrin beta (Fragment)	1.874	Up	1.19E-02
U3IAG4	Optineurin (Fragment)	1.916	Up	5.82E-03
U3J6G0	Histone H2A	1.926	Up	3.28E-02
U3IWS8	Hexokinase2	1.928	Up	6.89E-03
U3J989	"Beta-1,4-galactosyltransferase 1 (Fragment) "	1.930	Up	3.44E-03
U3J778	SAP domain-containing ribonucleoprotein (Fragment)	1.939	Up	1.60E-02
U3IZH0	Transgelin-2 (Fragment)	1.941	Up	3.59E-03
U3ID07	Complement C1s subcomponent	1.946	Up	9.14E-03
U3IMY2	EH domain-containing protein 4 (Fragment)	1.955	Up	3.65E-03
R0KZL1	Calreticulin (Fragment)	1.956	Up	3.66E-03
U3I742	Alpha-2-macroglobulin (Fragment)	1.967	Up	1.51E-02
U3I6U1	Rho GDP-dissociation inhibitor 2 (Fragment)	1.970	Up	5.59E-03
U3I2Q9	Transporter associated with antigen processing 1	1.971	Up	6.43E-03
R0JRR9	Ras-related protein Rab-32 (Fragment)	1.978	Up	4.35E-03
U3HZR3	Interferon-induced very large GTPase 1 (Fragment)	1.994	Up	2.32E-02
U3IKF3	Complement component C8 beta chain (Fragment)	1.999	Up	3.52E-03
U3J0M7	Signal transducer and activator of transcription 1 (Fragment)	2.001	Up	2.49E-04
R0KRZ1	Protein LYRIC (Fragment)	2.007	Up	8.04E-03
U3IWC6	E3 ISG15--protein ligase HERC5 (Fragment)	2.011	Up	7.23E-03
R0L541	Peroxisomal proliferator-activated receptor A-interacting complex 285 kDa protein (Fragment)	2.018	Up	3.39E-04
U3I793	Formin-like protein 1	2.037	Up	1.85E-03
U3IRC3	Poly [ADP-ribose] polymerase	2.039	Up	3.03E-04
U3IIL1	Annexin	2.063	Up	1.35E-02
U3ICV6	Delta-1 crystallin	2.072	Up	4.75E-02
R0LYX6	Complement component receptor 1-like protein (Fragment)	2.073	Up	5.91E-03
U3I8R2	Complement C4 (Fragment)	2.080	Up	7.98E-03
U3IEV3	Tyrosine-protein kinase	2.081	Up	9.59E-03
R0JNZ4	Macrophage-capping protein (Fragment)	2.084	Up	1.15E-02
R0LGG0	Complement C1q subcomponent subunit A (Fragment)	2.087	Up	4.12E-03
U3J0P4	IgGFc-binding protein	2.097	Up	2.50E-04
U3IN55	Complement component C9 (Fragment)	2.100	Up	2.76E-03
P00706	Lysozyme C-3	2.124	Up	1.18E-03

R0LW73	Complement C1q subcomponent subunit B (Fragment)	2.125	Up	2.04E-03
R0JP17	Serpin B10 (Fragment)	2.126	Up	1.71E-02
U3I028	Sterile alpha motif domain-containing protein 9-like (Fragment)	2.137	Up	1.13E-03
U3J2T8	Microfibrillar-associated protein 5 (Fragment)	2.148	Up	3.37E-02
U3HZV7	Ovoinhibitor	2.149	Up	3.33E-03
U3I321	"2',3'-cyclic-nucleotide 3'-phosphodiesterase (Fragment) "	2.156	Up	1.13E-02
U3J3R4	Cap-specific mRNA (Nucleoside-2'-O-)-methyltransferase 1	2.158	Up	2.67E-03
U3J1F2	Protein S100	2.178	Up	1.66E-02
U3J0V8	Signal transducer and activator of transcription 1	2.179	Up	4.89E-04
R0K5F7	OTU domain-containing protein 4 (Fragment)	2.191	Up	3.03E-03
U3ITB1	Alpha-2-macroglobulin-like 1 (Fragment)	2.204	Up	1.66E-02
U3HZM3	Uncharacterized protein	2.212	Up	6.10E-03
A0A0M4S8A2	Double-stranded RNA-dependent protein kinase	2.216	Up	1.57E-03
U3I1F8	Hemopexin (Fragment)	2.218	Up	2.05E-02
R0LGA4	Poly [ADP-ribose] polymerase (Fragment)	2.220	Up	7.96E-06
S4SM19	ATP-dependent RNA helicase (Fragment)	2.222	Up	3.83E-03
U3I7F0	MHC-linked complement C4	2.224	Up	3.24E-02
R0LEU9	"Chromosome 17 SCAF14597, whole genome shotgun sequence "	2.229	Up	3.25E-03
U3IXR6	Phospholipase D1	2.238	Up	2.60E-02
U3I6G9	Opioid growth factor receptor (Fragment)	2.255	Up	4.98E-04
U3IGY9	"Solute carrier family 2, facilitated glucose transporter member 6 (Fragment) "	2.329	Up	4.76E-03
Q2VQY9	MHC class I antigen alpha chain	2.423	Up	1.19E-02
A0A0B5JQU7	Viperin	2.488	Up	2.82E-02
U3INW9	Connective tissue growth factor (Fragment)	2.515	Up	4.69E-02
U3IKH3	Complement component C8 alpha chain (Fragment)	2.526	Up	2.58E-03
U3IRY0	Vimentin	2.528	Up	7.76E-03
Q14U75	Beta-2-microglobulin	2.533	Up	5.64E-03
A0A0M3RQ94	Retinoic acid inducible protein I	2.565	Up	7.15E-03
U3I8M2	Interferon-induced 35 kDa protein	2.578	Up	7.36E-03
U3IYM9	26S proteasome non-ATPase regulatory subunit 3	2.581	Up	2.32E-03
U3IEV1	Nicotinamide phosphoribosyltransferase (Fragment)	2.588	Up	4.20E-03
A0A023NCT5	Melanoma differentiation associated protein-5	2.593	Up	3.23E-02
U3I8Y0	Normal mucosa of esophagus-specific 1	2.596	Up	2.20E-02

	(Fragment)			
U3IB49	Leukocyte cell-derived chemotaxin 2	2.648	Up	5.63E-04
U3IGN0	N-myc-interactor (Fragment)	2.676	Up	1.73E-03
R0LDN0	Deleted in malignant brain tumors 1 protein	2.689	Up	7.45E-04
U3J2R0	Vitronectin (Fragment)	2.798	Up	6.82E-04
P00705	Lysozyme C-1	2.865	Up	7.37E-04
U3J8T9	L-amino-acid oxidase (Fragment)	2.903	Up	4.11E-03
U3ITA4	Argininosuccinate synthase	2.921	Up	5.94E-03
U3J802	"Spectrin beta chain, erythrocytic (Fragment) "	2.939	Up	2.28E-02
U3ITN8	Erythrocyte membrane protein band 4.2 (Fragment)	3.245	Up	2.69E-02
R0JBX5	Homeodomain-only protein (Fragment)	3.257	Up	3.45E-03
R0JXF2	Putative ATP-dependent RNA helicase DDX60 (Fragment)	3.276	Up	1.03E-02
R0LF52	Leukocyte elastase inhibitor	3.314	Up	1.97E-02
U3INH5	Complement component C7 (Fragment)	3.321	Up	8.83E-05
U3J4V2	Tubulin beta-6 chain (Fragment)	3.354	Up	2.16E-02
R0JS12	Serine protease inhibitor A3M (Fragment)	3.404	Up	9.15E-03
U3IA23	Fibrinogen gamma chain (Fragment)	3.493	Up	1.26E-03
U3I646	Carbonic anhydrase 2 (Fragment)	3.601	Up	2.94E-02
U3IF32	Ankyrin-1 (Fragment)	3.639	Up	2.27E-02
U3IY90	Serpin B10 (Fragment)	3.722	Up	3.06E-03
R0LJZ9	"UMP-CMP kinase 2, mitochondrial (Fragment) "	3.734	Up	3.20E-04
P02114	Hemoglobin subunit beta	3.794	Up	1.17E-02
R0JKP4	Aquaporin-3 (Fragment)	3.817	Up	3.72E-02
R0KUN6	Protein-glutamine gamma-glutamyltransferase E (Fragment)	3.945	Up	2.94E-04
U3IJS3	Ceruloplasmin (Fragment)	4.045	Up	2.84E-03
R0JSX9	Fibrinogen alpha chain (Fragment)	4.182	Up	3.95E-02
R0JS80	Fibrinogen beta chain (Fragment)	4.367	Up	1.11E-03
U3IL29	Cytochrome P450 2C1 (Fragment)	4.377	Up	1.71E-02
R0K9R1	BCL2-like protein 15 (Fragment)	4.378	Up	2.41E-03
P04442	Hemoglobin subunit alpha-D	4.518	Up	8.94E-03
P02740	Serum amyloid A protein	4.531	Up	1.00E-02
R0KHM3	Interferon-induced protein with tetratricopeptide repeats 5 (Fragment)	4.664	Up	4.08E-02
U3IRN3	Protein MRP-126	4.757	Up	3.07E-03
C7EKN9	Hemoglobin alpha A subunit	4.958	Up	1.25E-02
R0LE74	Histone H5 (Fragment)	5.236	Up	1.25E-02
R0JE94	Interferon alpha-inducible protein 6 (Fragment)	5.548	Up	8.58E-03
U3IC83	Serum amyloid A protein	5.698	Up	5.21E-04
U3I466	Alpha-1-acid glycoprotein	5.703	Up	2.40E-02

Table S4 The differential expressed proteins between CDRV and NDRV infections.

Protein accession	Protein description	CDRV vs NDRV Ratio	Regulated Type	P value
C7EKN9	Hemoglobin alpha A subunit	0.354	Down	7.94E-03
P02114	Hemoglobin subunit beta	0.385	Down	7.40E-03
P04442	Hemoglobin subunit alpha-D	0.375	Down	4.47E-03
R0JKP4	Aquaporin-3 (Fragment)	0.373	Down	4.49E-02
R0JS80	Fibrinogen beta chain (Fragment)	0.400	Down	1.25E-04
R0JSX9	Fibrinogen alpha chain (Fragment)	0.369	Down	3.18E-02
R0KF58	Transmembrane protein 85	1.558	Up	4.47E-02
R0L854	"Nuclease EXOG, mitochondrial (Fragment) "	1.520	Up	4.29E-02
R0LCK1	Dipeptidyl-peptidase 1 (Fragment)	1.598	Up	2.46E-03
R0LE74	Histone H5 (Fragment)	0.329	Down	9.25E-03
R0LF52	Leukocyte elastase inhibitor	0.509	Down	4.89E-02
R0LQT9	Lymphoid-specific helicase (Fragment)	0.617	Down	1.65E-02
U3HZM3	Uncharacterized protein	0.580	Down	4.58E-03
U3I539	von Willebrand factor (Fragment)	0.645	Down	3.20E-02
U3I646	Carbonic anhydrase 2 (Fragment)	0.440	Down	3.99E-02
U3IA23	Fibrinogen gamma chain (Fragment)	0.426	Down	1.48E-03
U3IB49	Leukocyte cell-derived chemotaxin 2	0.632	Down	3.14E-04
U3IF32	Ankyrin-1 (Fragment)	0.406	Down	2.86E-02
U3ILM0	Protein phosphatase PTC7 (Fragment)	1.605	Up	4.70E-02
U3INH5	Complement component C7 (Fragment)	0.550	Down	2.92E-0

				4
U3IRV5	Galectin	1.521	Up	1.09E-02
U3ITN8	Erythrocyte membrane protein band 4.2 (Fragment)	0.459	Down	3.18E-02
U3IVH5	Ribonuclease CL2 (Fragment)	1.521	Up	2.44E-02
U3IWQ9	Vitamin K-dependent protein S (Fragment)	0.635	Down	4.26E-03
U3IY90	Serpin B10 (Fragment)	0.583	Down	2.85E-03
U3J0P4	IgGFc-binding protein	0.503	Down	4.11E-03
U3J2R0	Vitronectin (Fragment)	0.486	Down	1.31E-04
U3J370	SUN domain-containing protein 2 (Fragment)	0.585	Down	6.74E-03
U3J4V2	Tubulin beta-6 chain (Fragment)	0.427	Down	2.17E-02
U3J6G0	Histone H2A	0.540	Down	3.96E-02
U3J778	SAP domain-containing ribonucleoprotein (Fragment)	0.664	Down	3.83E-02
U3J802	"Spectrin beta chain, erythrocytic (Fragment) "	0.425	Down	3.11E-02

Table S5 KEGG enrichment analysis of the DEPs under CDRV infection.

KEGG pathway	Mapping	Background	All Mapping	All Background	Fold enrichment	Fisher' exact test p value
apla01100 Metabolic pathways	55	640	113	2200	1.67	8.925E-06
apla01200 Carbon metabolism	14	83	113	2200	3.28	0.0001994
apla05168 Herpes simplex infection	11	78	113	2200	2.75	0.0052525
apla00620 Pyruvate metabolism	6	26	113	2200	4.49	0.0087098
apla00500 Starch and sucrose metabolism	5	18	113	2200	5.41	0.0111891
apla02010 ABC transporters	5	19	113	2200	5.12	0.0136266
apla00983 Drug metabolism - other enzymes	5	24	113	2200	4.06	0.0307219
apla01212 Fatty acid metabolism	6	39	113	2200	3	0.0451295

Table S6 KEGG enrichment analysis of the DEPs under NDRV infection.

KEGG pathway	Mapping	Background	All Mapping	All Background	Fold enrichment	Fisher' exact test p value
apla01100 Metabolic pathways	63	640	138	2200	1.57	2.461E-05
apla01200 Carbon metabolism	17	83	138	2200	3.27	3.027E-05
apla00010 Glycolysis / Gluconeogenesis	10	37	138	2200	4.31	0.0003014
apla04145 Phagosome	14	81	138	2200	2.76	0.0011451
apla00030 Pentose phosphate pathway	7	22	138	2200	5.07	0.0017004
apla01230 Biosynthesis of amino acids	10	50	138	2200	3.19	0.0030263
apla00500 Starch and sucrose metabolism	6	18	138	2200	5.31	0.0038561
apla00620 Pyruvate metabolism	7	26	138	2200	4.29	0.0042659
apla05168 Herpes simplex infection	12	78	138	2200	2.45	0.0076901
apla05164 Influenza A	11	70	138	2200	2.51	0.0099832
apla00061 Fatty acid biosynthesis	4	9	138	2200	7.09	0.0150345
apla00052 Galactose metabolism	5	17	138	2200	4.69	0.0181365
apla02010 ABC transporters	5	19	138	2200	4.2	0.0268143
apla00051 Fructose and mannose metabolism	5	21	138	2200	3.8	0.0376047

Table S7 The result data of PRM verification.

Protein Accession	Peptide	Average Measured Retention Time	CDRV-1 Normalized Area	CDRV-2 Normalized Area	CDRV-3 Normalized Area	CON-1 Normalized Area	CON-2 Normalized Area	CON-3 Normalized Area	NDRV-1 Normalized Area	NDRV-2 Normalized Area	NDRV-3 Normalized Area
U3IRV5	FDCHGDVNTIVCNSK	22.72	4.17E+07	4.52E+07	4.34E+07	4.64E+07	2.55E+07	2.16E+07	3.78E+07	3.13E+07	2.98E+07
A0A0C4K5J8	EEVSIPLYPPSR	38.01	3.01E+08	3.24E+08	2.39E+08	3.52E+07	3.38E+07	2.11E+07	2.57E+08	2.54E+08	3.45E+08
A0A0M4S8A2	NIEPIGEGGFGNVFK	45.3	1.28E+08	1.52E+08	1.46E+08	2.25E+07	3.05E+07	2.32E+07	5.84E+07	1.28E+08	1.61E+08
U3I8M2	GEVTNIQFQPSR	27.66	1.18E+08	1.47E+08	1.53E+08	2.64E+07	3.53E+07	2.91E+07	2.34E+08	1.80E+08	2.01E+08
U3J0V8	NSVIDVEQDIK	32	1.22E+08	1.03E+08	1.45E+08	2.01E+07	2.92E+07	2.49E+07	1.12E+08	1.22E+08	1.52E+08
U3J0V8	TLEDVQDEYDFK	33.32	1.47E+08	1.11E+08	1.94E+08	2.85E+07	3.61E+07	3.54E+07	1.08E+08	1.35E+08	1.89E+08
R0KHM3/W8GPS8	TPNLPYFLR	45.09	1.30E+08	1.91E+08	1.37E+08	2.62E+06	8.14E+06	2.28E+06	1.30E+08	5.56E+07	2.38E+08
R0KHM3/W8GPS8	NPDNEEYLSALCELR	45.46	3.05E+07	4.29E+07	3.60E+07	1.26E+06	3.13E+06	1.57E+06	4.57E+07	2.11E+07	7.64E+07
R0L013/U3IBH0	VAIEVICENVGR	34.1	8.10E+07	7.65E+07	9.90E+07	2.38E+07	2.69E+07	3.84E+07	7.71E+07	1.01E+08	1.18E+08
R0L013/U3IBH	IIAANPLNLK	35.37	7.95E+07	7.16E+07	7.38E+07	1.37E+07	1.58E+07	2.19E+07	6.32E+07	8.90E+07	9.19E+07

0											
P02740	ADQEANAWGR	16.98	2.26E+08	1.43E+08	2.64E+08	1.90E+05	2.06E+06	1.37E+06	1.26E+08	2.03E+08	1.41E+08
U3IGV1/R0JR24	LPPNVVAEPDLLK	39.36	5.50E+08	6.61E+08	7.34E+08	7.95E+08	6.68E+08	5.28E+08	3.37E+08	6.51E+08	5.72E+08
U3IGV1/R0JR24	GVDEGPDGLR	17.73	7.90E+08	1.05E+09	1.19E+09	1.83E+09	1.03E+09	9.36E+08	8.02E+08	1.05E+09	1.02E+09
U3IHG8	ALSDIAQR	17.79	7.31E+09	9.38E+09	1.09E+10	2.31E+10	1.13E+10	1.55E+10	4.35E+09	8.65E+09	7.43E+09
U3IHG8	INVENTEENR	13.83	2.60E+08	3.22E+08	4.25E+08	6.30E+08	4.98E+08	6.70E+08	1.65E+08	3.07E+08	3.04E+08
U3I0F9	GSGTAEVELK	16.53	8.39E+07	4.61E+07	8.37E+07	3.60E+07	4.50E+07	4.24E+07	1.17E+08	1.17E+08	9.62E+07
U3I0F9	CDENVLWLDYK	44.41	3.11E+07	1.55E+07	4.05E+07	8.93E+06	1.37E+07	1.35E+07	3.28E+07	3.28E+07	2.82E+07
U3J925	TIAIIAEGIPEALTR	48.64	3.67E+08	1.04E+09	7.15E+08	2.90E+09	2.40E+09	2.36E+09	2.31E+08	6.65E+08	7.92E+08
U3J925	DLVSSLTSGLLTIGDR	55.7	1.71E+08	4.30E+08	2.85E+08	1.26E+09	1.00E+09	8.85E+08	9.44E+07	2.38E+08	3.56E+08
U3ICK7	ELLNIPENYK	35.52	4.93E+08	2.78E+08	4.64E+08	8.51E+08	5.33E+08	5.26E+08	2.39E+08	1.88E+08	2.61E+08
U3ICK7	EDLLGFALK	47.3	3.74E+08	2.21E+08	3.39E+08	8.90E+08	4.83E+08	5.06E+08	9.90E+07	1.37E+08	1.51E+08
U3ISI9/R0L1A8	LGGSEDEQIQNACDK	17.04	8.99E+07	1.28E+08	1.10E+08	2.50E+08	1.76E+08	2.25E+08	4.95E+07	1.00E+08	1.16E+08
U3ISI9/R0L1A8	LFVLFGAEILK	53.17	2.03E+08	3.13E+08	3.27E+08	6.73E+08	5.32E+08	6.34E+08	9.74E+07	2.68E+08	3.05E+08

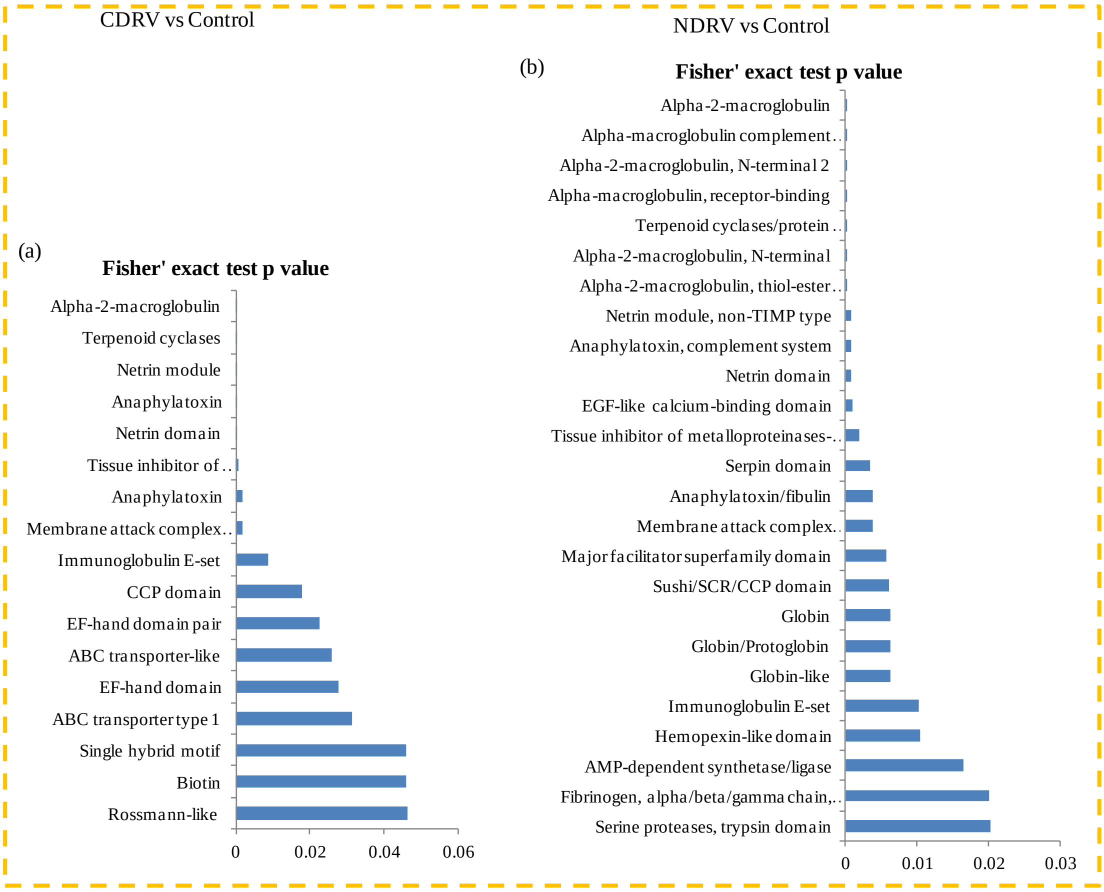

Figure S1 **Protein domain enrichment analysis the DEPs under C/NDRV infections.** (a) Protein domain enrichment analysis the DEPs under CDRV infections. (b) Protein domain enrichment analysis the DEPs under NDRV infections.
